# A stable JAZ protein from peach mediates the transition from outcrossing to self-pollination

**DOI:** 10.1186/s12915-015-0124-6

**Published:** 2015-02-13

**Authors:** Sherif Sherif, Islam El-Sharkawy, Jaideep Mathur, Pratibha Ravindran, Prakash Kumar, Gopinadhan Paliyath, Subramanian Jayasankar

**Affiliations:** Vineland Research Station, Department of Plant Agriculture, University of Guelph, 4890 Victoria Av. N, P.O. Box 7000, Vineland Station, ON L0R 2E0 Canada; Department of Plant Agriculture, University of Guelph, 50 Stone Road East, Guelph, Ontario N1G2W1 Canada; Department of Horticulture, Faculty of Agriculture, Damanhour University, Al-Gomhuria St, PO Box 22516, Damanhour, Al-Behira Egypt; Department of Molecular and Cellular Biology, University of Guelph, 50 Stone Road East, Guelph, Ontario N1G2W1 Canada; Department of Biological Sciences, National University of Singapore, Science Drive 4, Singapore, 117543 Singapore

**Keywords:** JAZ proteins, Jasmonic acid, Autogamy, Cleistogamy, Peach, Floral display

## Abstract

**Background:**

Variations in floral display represent one of the core features associated with the transition from allogamy to autogamy in angiosperms. The promotion of autogamy under stress conditions suggests the potential involvement of a signaling pathway with a dual role in both flower development and stress response. The jasmonic acid (JA) pathway is a plausible candidate to play such a role because of its involvement in many plant responses to environmental and developmental cues. In the present study, we used peach (*Prunus persica* L.) varieties with showy and non-showy flowers to investigate the role of JA (and JA signaling suppressors) in floral display.

**Results:**

Our results show that PpJAZ1, a component of the JA signaling pathway in peach, regulates petal expansion during anthesis and promotes self-pollination. *PpJAZ1* transcript levels were higher in petals of the non-showy flowers than those of showy flowers at anthesis. Moreover, the ectopic expression of *PpJAZ1* in tobacco (*Nicotiana tabacum* L.) converted the showy, chasmogamous tobacco flowers into non-showy, cleistogamous flowers. Stability of PpJAZ1 was confirmed *in vivo* using PpJAZ1-GFP chimeric protein. PpJAZ1 inhibited JA-dependent processes in roots and leaves of transgenic plants, including induction of JA-response genes to mechanical wounding. However, the inhibitory effect of PpJAZ1 on JA-dependent fertility functions was weaker, indicating that PpJAZ1 regulates the spatial localization of JA signaling in different plant organs. Indeed, JA-related genes showed differential expression patterns in leaves and flowers of transgenic plants.

**Conclusions:**

Our results reveal that under stress conditions – for example, herbivore attacks -- stable JAZ proteins such as PpJAZ1 may alter JA signaling in different plant organs, resulting in autogamy as a reproductive assurance mechanism. This represents an additional mechanism by which plant hormone signaling can modulate a vital developmental process in response to stress.

**Electronic supplementary material:**

The online version of this article (doi:10.1186/s12915-015-0124-6) contains supplementary material, which is available to authorized users.

## Background

The transition from high levels of outcrossing (allogamy) to predominantly self-fertilization (autogamy) represents a dramatic evolutionary change in the reproductive biology of many angiosperms [1]. The allocation of resources in floral anatomy was profoundly altered with the shift to ‘selfing’: compared to their allogamous counterparts, autogamous flowers tend to produce less pollen, scent and nectar, have a shorter distance between receptive stigma and dehiscent anthers, and have smaller petals that open less at maturity [2,3]. One explanation for the shift to selfing is the evolutionary advantage that would accrue to selfing individuals over outcrossers when pollinators or mating partners are scarce, generally referred to as ‘reproductive assurance’ [4]. In some mixed mating systems, the production of cleistogamous flowers (obligatory selfing) is promoted in stressed environments [5]. Earlier studies also have shown that the application of plant hormones that mimic sustained growth conditions (that is, gibberellic acid (GA)) encourages the production of chasmogamous flowers [6], while the application of hormones that mimic water stress (that is, abscisic acid (ABA)) promotes cleistogamy [7]. These findings draw attention to internal signaling pathways that may have been key instruments in the evolution of selfing through their dual role in environmental stress response and flower development. Methyl jasmonate (MeJA) and other members of the jasmonate family of compounds (referred to as JAs), represent a major group of plant hormones that could play such a role, as the importance of JA in mediating plant responses to abiotic and biotic stimuli, as well as its contribution to different aspects of flower development, is well established (see reviews: [8-11]).

The role of JA in flower development has been correlated primarily with male fertility, as most *Arabidopsis* JA-biosynthesis and JA-signaling mutants show defects in stamen development [8,12-15]. However, components of JA biosynthesis and signaling can affect other aspects of reproductive development in plant species. For instance, mutations in *Coronatine Insensitive 1* (*COI1*), an integral component of JA perception in *Arabidopsis*, results in male sterility, while mutations in the *COI1* homolog in tomato results in female sterility due to defects in the maternal control of seed maturation [16]. In addition, *Arabidopsis* JA-biosynthesis mutants such as OPDA reductase 3 (*opr3*) and allene oxide synthase (*aos*) are male sterile due to inviable pollen or insufficient filament elongation [8]; whereas the inhibition of JA production in maize JA-biosynthesis mutants Tasselseed 1 (*ts1*), *opr7* and *opr8* has negative impacts on male sex determination, as staminates are converted to pistillate flowers in these mutants [17,18]. In rice, defects in JA biosynthesis or perception proteins leads to altered spikelet morphology; floral organ identity and number [19]; flower opening and closure; as well as anther dehiscence [20]. These mutant phenotypes demonstrate that JA biosynthesis and signaling elements are necessary for proper flower development but that the roles of JA elements have diversified during the evolution of flowering plants.

Positive and negative feedback loops regulating JA production and function are primarily coordinated through the degradation and synthesis/stabilization of JA suppressor proteins, collectively known as Jasmonate ZIM-Domain (JAZ) proteins. The degradation of JAZ proteins is mediated by the F-box protein COI1, which associates with other partners (SKP1 and Cullin) of E3 ubiquitin ligase to form SCF^COI1^ [15,21]. Upon perception of the JA signal, SCF^COI1^ targets JAZ proteins for ubiquitination and subsequent degradation by the 26S proteasome [15,21,22]. The interaction between JAZ and COI1 constitutes the co-receptor complex required for the perception of the bioactive hormone, JA-isoleucine (JA-Ile) [23]. The degradation of JAZ proteins unblocks the activity of transcription factors responsible for anthocyanin biosynthesis and trichome initiation (that is, MYB75 and GL3) [24]; stamen development (that is, MYB21 and MYB24) [25]; resistance to herbivores (that is, MYC3 and MYC4) [26,27] and resistance to necrotrophic fungi (that is, EIN3) [28]. As a negative feedback mechanism, *JAZ* genes are transcriptionally upregulated in response to high levels of JA [21]. Among these genes, alternative splice variants (that is, JAZ10.4) are immune to degradation and repress the JA pathway in the presence of hormone [29]. Although the general role of JAZ proteins during flower development can be anticipated based on their inhibition of the JA signal transduction pathway, the diverse phenotypes associated with JA-insensitivity in different plant species suggest species-specific functions for JAZ proteins.

In this study, we used peach (*Prunus persica* L.) varieties producing either showy or non-showy flowers in order to investigate the role of JA pathway components in floral display. Unlike other stone fruits, such as plums, cherries and apricots, which all require cross-pollination in order to achieve fruit set, most peach varieties are self-fertile [30]. Therefore, these peach varieties, with their distinct flower appearance, present a good model to study JA and JAZ roles in petal morphology, independent of the role JA plays in male or female fertility. Our results show that a member of the JAZ family, PpJAZ1, regulates flower development through controlling petal elongation and flower opening, a previously uncharacterized role for the JAZ proteins.

## Results

### Showy versus non-showy peach flowers

**Figure 1:**
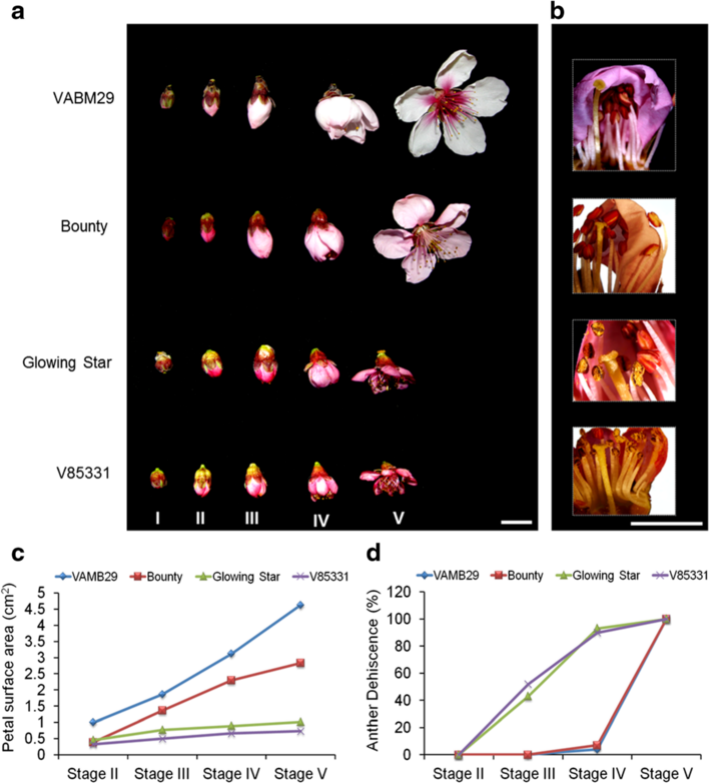
**Different patterns of flower opening, petal growth and anther dehiscence among peach varieties. (a)** Four peach varieties were used in this study as representatives for showy, ‘VABM29’ and ‘Bounty’, and non-showy, ‘Glowing Star’ and ‘V85331’ flowers. Flowers and flower buds were collected in five stages based on the development of outer and inner floral organs, scale bar *=* 20 mm. **(b)** Anther dehiscence and pollen release were monitored in stage III of flower development. Micrographs show closed flowers with three petals removed from each flower to show the development of stamen, scale bar = 6 mm. Petal growth, represented as petal surface area **(c)** and anther dehiscence (%) **(d)** were recorded for the four peach varieties during four stages of flower development (n = 12).

To elucidate the molecular basis of flower opening and selfing in peach, we examined two varieties with showy flowers (‘VABM29’ and ‘Bounty’) and two varieties with non-showy flowers (‘Glowing Star’ and ‘V85331’) (Figure 1a). There were only minor differences among the genotypes in terms of stamen and pistil lengths during various stages of flower development. In contrast, clear differences were found among varieties in terms of petal surface area and the timing of anther dehiscence. Petal surface area was significantly different (up to fourfold) between the showy and non-showy flowers, particularly after stage III (Figure 1c). Cell size measurements indicated that reduced petal growth of the non-showy flowers is due to reduced cell expansion (see Additional file 1: Figure S1). Dehiscent anthers were observed as early as stage III for the non-showy flowers, and not until stage IV in the showy varieties (Figure 1b,d). Up to 40% of anthers dehisced in ‘Glowing Star’ and ‘V85331’ in closed buds (stage III), whereas in ‘VABM29’ and ‘Bounty’ no more than 5% of anthers dehisced at stage IV, before the number dramatically jumped to 100% at anthesis (stage V) (Figure 1d). Applying conventional botanical nomenclature, the non-showy genotypes were thus considered to be pre-anthesis cleistogamous (pCL) while the showy genotypes were considered chasmogamous (CH).

### JA-dependent regulation of floral display in peach

**Figure 2:**
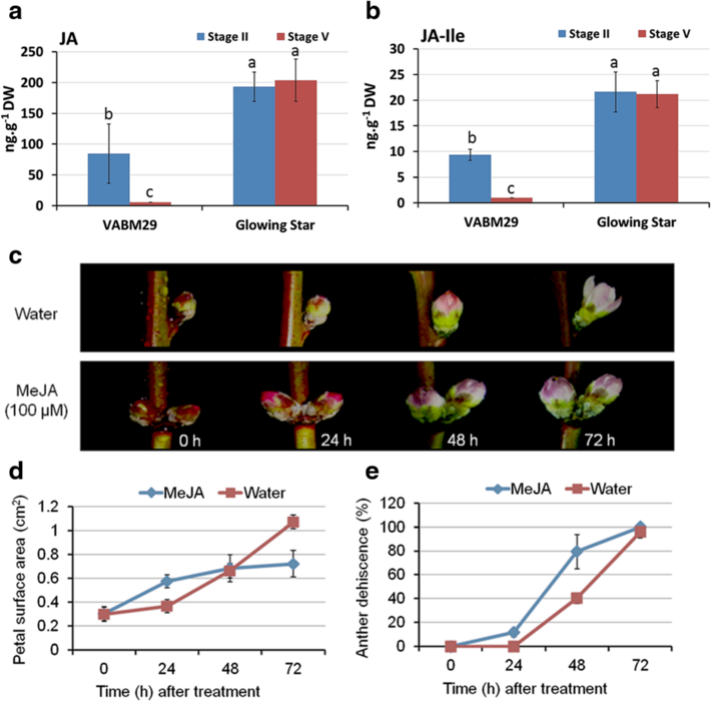
**Quantification and effect of JAs during flower opening in peach. (a-b)** Total JAs and JA-Ile were quantified in petals of ‘VABM29’ and ‘Glowing star’ at 0% (Stage II) and at 100% anther dehiscence (Stage V). Values are the mean and standard error of three biological replicates. Means in each column having the same letter are not significantly different (*P* <0.05, Tukey-Kramer HSD test). **(c)** MeJA (100 μM) or water was sprayed on ‘Glowing Star’ flowers (Stage II) and pictures were captured for the same flower buds at 0, 24, 48 and 72 hours after treatment. **(d-e)** Measurements for petal surface area (cm2) **(d)** and anther dehiscence (%) **(e)** were recorded (n = 12) at the designated times. JA, jasmonic acid; JA-Ile, JA-isoleucine; MeJA, methyl jasmonate.

To examine whether the observed differences among peach genotypes can be explained by changes in jasmonates biosynthesis levels, the endogenous levels of JA and JA-Ile were quantified in petals of ‘VABM29’ and ‘Glowing Star’ at stage II (0% anther dehiscence) and stage V (100% anther dehiscence). Given the potential role of stamens as a source of JA [13,31], the selection of these two stages (II and V) was meant to avoid any residual effect the non-dehiscent stamens might have on the level of JA and JA-Ile in petals. As shown in Figure 2a, b, both JA and JA-Ile were significantly more abundant in petals of ‘Glowing Star’ at both developmental stages. While the levels of JA and JA-Ile declined significantly in ‘VABM29’ at stage V, no differences were noticed between the two developmental stages in ‘Glowing Star’ (Figure 2a,b). Interestingly, the expression levels of two JA biosynthesis genes, *PpOPR3* and *PpLOX3*, encoding for OPDA-reductase 3 and lipoxygenase 3 (13-LOX), respectively, did not follow the same pattern of JA and JA-Ile levels in petals. Although the expression of both genes was significantly higher in pCL varieties at stage II, transcript levels declined almost equally in all varieties at anthesis (Stage V) (see Additional file 2: Figure S2). These results parallel a recently published report concerning the regulation of JA pools during the development of coyote tobacco (*Nicotiana attenuata*) flowers, in which the authors demonstrated that transcriptional activation of JA-biosynthesis genes precedes rapid flower maturation and accumulation of JA-Ile [32].

These results together indicate that increased JA concentration in flower buds may inhibit full petal growth, a hypothesis partially supported by the application of MeJA (100 μM) to stage II flowers of ‘Glowing Star’ (Figure 2c). MeJA treatment accelerated petal growth during the first 48 hours, but growth ceased 72 hours after treatment and most flowers failed to open. By contrast, petals of water-treated flowers expanded only slightly during the first 48 hours, but increased dramatically in size by 72 hours post-treatment, with an average increase of 3 mm^2^ more than the MeJA-treated flowers (Figure 2d). Such differences in petal growth rate were accompanied by variations in the percentage of dehisced anthers, which significantly increased (*P* <0.01) after 24 and 48 hours of MeJA treatment compared to water treatment (Figure 2e). The application of MeJA to VABM29 did not, however, affect the ratio of anther dehiscence or the petal surface area compared to water treatment (data not shown), suggesting that differences among pCL and CH varieties cannot be explained by components of the JA biosynthesis pathway only.

### The expression profile of peach JAZ genes

**Figure 3:**
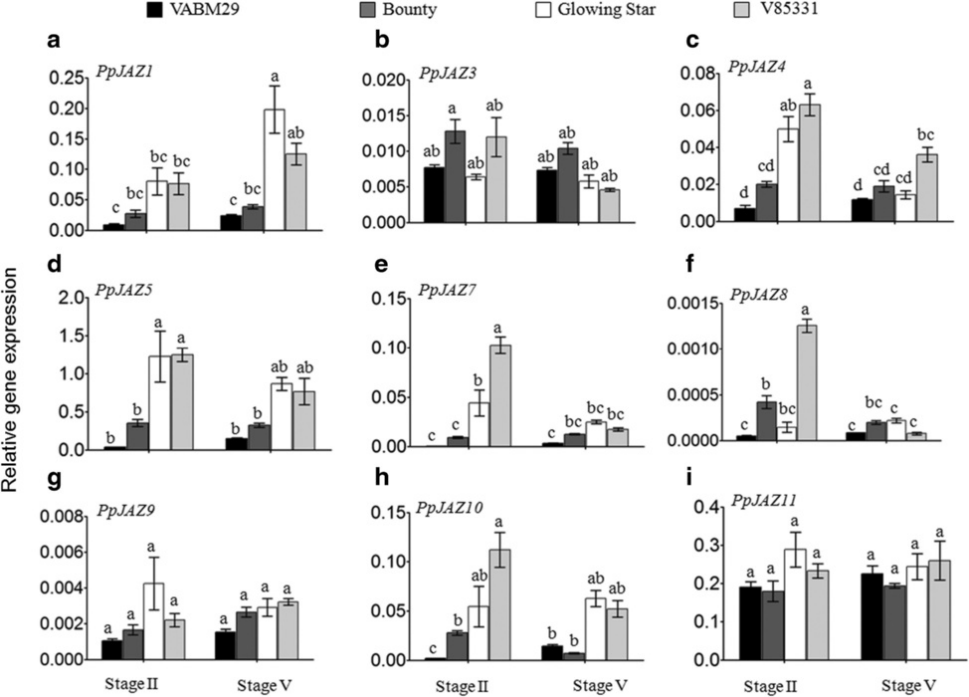
**Differential expression of various** ***PpJAZ*** **genes in petals of showy and non-showy peach varieties. (a-i)** The gene expression was quantified in petals of ‘VABM29’, ‘Bounty’, ‘Glowing Star’ and ‘V85331’ at stages II (0% anther dehiscence) and stage V (100% anther dehiscence). The expression of each gene was calculated relative to the expression of peach actin (*PpActin*) gene in each sample. Bars represent the mean of three biological replicates ± SE. Means in each column having the same letter are not significantly different (*P* <0.05, Tukey-Kramer HSD test). HSD, honest significant differenc; SE, standard error.

To gain insight into the role of JA signaling components in the regulation of pCL versus CH phenotypes of peach flowers, nine *JAZ* genes were isolated from a peach cDNA library and their expression levels were monitored during the transition of flowers from the bud stage to anthesis. Peach JAZ proteins (PpJAZs) contained the characteristic ZIM domain and Jas motif that are necessary for most JAZ functions (see Additional file 3: Text S1). Based on the conserved motifs and on phylogenetic analysis using the maximum likelihood bootstrap method, the nine identified peach JAZ proteins were assigned to seven classes (see Additional file 4: Figure S3). Transcript levels of five peach *JAZ* genes (*PpJAZ4*, *PpJAZ5*, *PpJAZ7*, *PpJAZ8* and *PpJAZ10*) followed the same pattern as JA-biosynthesis genes *PpOPR3* and *PpLOX3* in flower petals, wherein transcripts were significantly more abundant in pCL compared to CH varieties at stage II and then declined dramatically in all varieties at stage V (Figure 3c,d,e,f and h). Meanwhile, three *JAZ* genes (*PpJAZ3*, *PpJAZ9* and *PpJAZ11*) had constant expression levels before and after flower opening (Figure 3b, g and i). *PpJAZ1* was the only JAZ member showing higher expression in stage V than in stage II and in pCL compared to CH varieties (Figure 3a), which strongly suggests a distinct role for this gene during anthesis. The promoter region (1,230 bp upstream of the translational start site) of *PpJAZ1* was isolated from genomic libraries of ‘VABM29’, ‘Bounty’, ‘Glowing Star’ and ‘V85331’. The promoter regions contained a number of predicted binding sites for transcription factors with demonstrated roles in floral pigmentation (that is, MYB-homologous B), petal growth (that is, MYB.Ph3) and floral development (that is, Zinc finger-homeodomain genes) (see Additional file 5: Text S2 and Additional file 6: Table S1). It is also worth noting that one of the *cis*-regulatory elements that is present in the promoter of *PpJAZ1* is the binding site for the floral homeotic protein, AGAMOUS, which interferes with JA biosynthesis during late stages of stamen development through its binding to the promoter of the DEFECTIVE IN ANTHER DEHISCENCE1 (DAD1), a catalytic enzyme of JA [31]. Although all the above-mentioned *cis*-acting elements exist in pCL and CH varieties, a single nucleotide polymorphism (SNP) was detected in the binding site of AINTEGUMENTA transcription factor, which is involved in regulation of cell division and organ size [33]. Interestingly, this SNP is present in the sequence retrieved from ‘VAMB29’ which has the largest showy flower among all the studied varieties. Further investigations on the role of these *cis*- and *trans*-acting elements on the expression of *PpJAZ1* are likely to unravel additional molecular elements in JA signaling and action during flower development.

### PpJAZ1, a cleistogamy-inducing gene

**Figure 4:**
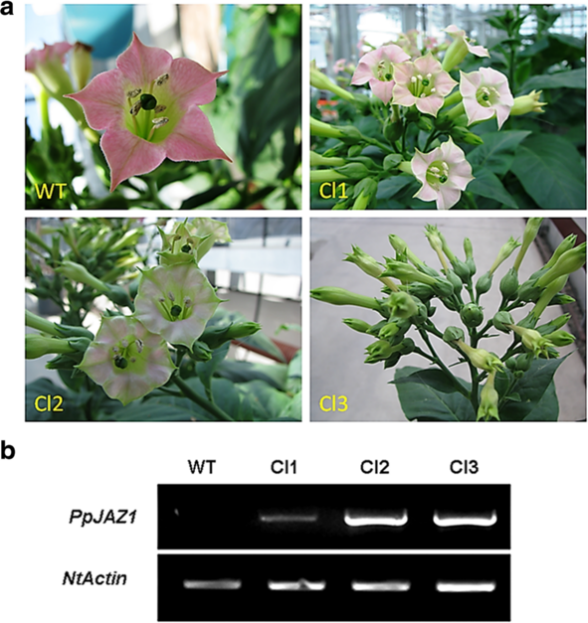
**Ectopic expression of** ***PpJAZ1*** **in tobacco alters corolla pigmentation and flower opening leading to cleistogamy** ***.*** **(a)** Three T0 transgenic tobacco plants were identified and selected from independent transformation events. While Cl1 did not show any major differences compared to WT except for having less corolla pigmentation, Cl2 and Cl3 plants were partially or completely closed, respectively. **(b)** The expression of *PpJAZ1* transgene in transgenic lines was quantified relative to that of *NtActin*. WT, wild type.

**Figure 5:**
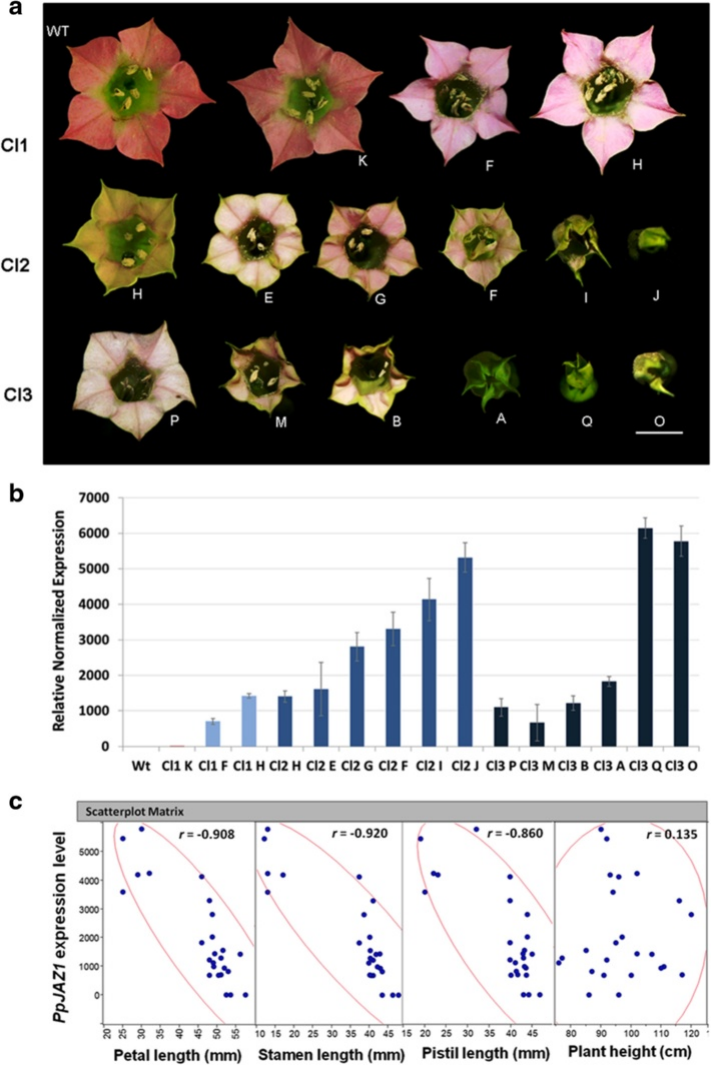
**T3 transgenic tobacco plants showed diverse patterns of flower opening. (a)** Compared to WT tobacco flowers, flowers of transgenic tobacco plants had less corolla coloration, less petal elongation at anthesis, showed typical cleistogamy phenotype or had impaired flower buds. Flowers were collected from T3 tobacco plants at the anthesis stage or shortly after anther dehiscence for those flowers that did not open. Flowers from each transgenic group were arranged in three panels representing three groups of transgenic lines (Cl1, Cl2 and Cl3) generated from three independent T0 plants. Inside each group the lines were arranged in the same way present in the X-axis of graph b. **(b)** The level of *PpJAZ1* transgene was quantified in leaves of each transgenic line using the qRT-PCR approach. The gene expression in each line was normalized to that of *NtActin* and was calculated relative to the gene expression of a reference sample (the one that had the lowest expression). The results represent the mean ± SE of three replicates prepared from different leaves of the same plant. **(c)** Scatter plot matrix generated based on the data recorded for 25 transgenic and three WT tobacco plants. r is calculated using Pearson correlation coefficient. WT, wild type.

**Figure 6:**
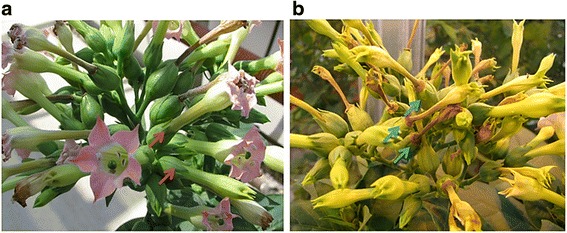
**Cleistogamous tobacco flowers set fruits as normal as the wild-type flowers.** While wild-type plants do not show any fruits in the closed-bud stage or in the recently fertilized flowers as indicated by the red arrows **(a)**, *PpJAZ1* tobacco from Cl3 A produced only cleistogamous flowers and set fruits as indicated by the blue arrows **(b)**, confirming the self-fertility nature of these flowers.

To test the functionality of *PpJAZ1* in the regulation of the non-showy appearance, we expressed the open reading frame (ORF) of *PpJAZ1* in tobacco (*Nicotiana tabacum* L., cv. PetH4), which carries typical showy CH flowers. It is worth noting that genetic transformation of peach is still in its infancy and it would take decades to generate stable transgenic trees. On the other hand, the tobacco transformation procedure is well established and transgenic plants/seeds can be relatively easily obtained. Three transgenic tobacco lines (Cl1, Cl2 and Cl3), selected from several independent transformation events at T0 (Figure 4), were selfed successively to generate T1 and T2 seeds. The segregation of T1 seeds on the selection medium did not follow the expected 3:1 Mendelian segregation, which along with the numerous floral phenotypes obtained in subsequent generations points to multiple transgene insertions. The level of *PpJAZ1* transgene, flower morphology parameters and other molecular investigations were done on T3 plants. Compared to the wild type (WT), flowers of transgenic plants showed different levels of petal pigmentation, size, flower opening and even fertility. In the Cl1 group, flowers lacked pigmentation, becoming whitish rather than rose-pink, but flower size and opening were little affected compared to WT (Figure 5a). In addition to the loss of petal pigmentation, flowers of the Cl2 and Cl3 groups were partially or completely closed (that is, Cl2 I, Cl3 M, Cl3 B and Cl3 A), showed defects in stamen morphology (that is, Cl2 F) or exhibited short stamen filaments combined with defects in flower opening (that is, Cl2 J, Cl3 O and Cl3 Q) (Figure 5a). The level of *PpJAZ1* transgene expression exhibited a near perfect correlation with defects in petal pigmentation and petal size of the same transgenic group (Figure 5b). In the 25 transgenic plants studied, the correlation between the level of *PpJAZ1* transgene and plant height or leaf width was weak (r = 0.135 and 0.159, respectively). However, the level of transgene was negatively correlated (r = - 0.908, - 0.920, - 0.860) with petal, stamen and pistil lengths, respectively (Figure 5c, Additional file 6: Table S2), collectively indicating that PpJAZ1 mainly affects floral morphology but not vegetative growth. Moreover, fruit set was not affected in most transgenic plants, even in the completely closed flowers (that is, Cl3 A) (Figure 6, Additional file 6: Table S2). It can be thus summarized that PpJAZ1 promotes self-fertilization in closed flower buds, which is effectively an induced cleistogamy.

### Features of JA-insensitivity in transgenic plants

**Figure 7:**
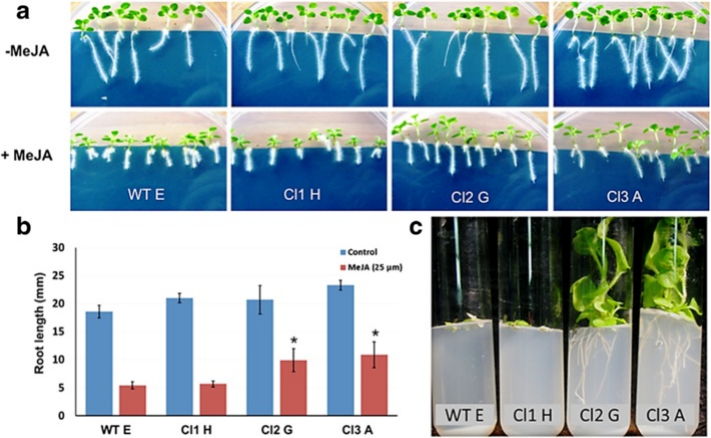
**JA insensitivity of tobacco plants expressing PpJAZ1. (a)** Seeds from WT, Cl1 H, Cl2 G and Cl3 A were grown on MS agar without (upper panel) and with 25 μM MeJA (lower panel) for 4 days at 4°C and then for 12 days at 25°C. The growth of Cl2 G and Cl3 A roots was less inhibited by MeJA (25 μM) compared to WT E and Cl1 H. **(b)** Values of root lengths are the means of three replicates (12 seedlings each) with SE. Genotypes marked with asterisk (*) are significantly higher than WT E, according to Tukey-Kramer HSD (*P* <0.05). **(c)** The effect of long-term exposure to JA on root length was examined by growing the seeds on MS medium supplied with MeJA (50 μM) for 40 days. Transgenic plants from Cl2 G and Cl3 A continued to grow on MeJA containing medium and produced normal roots and shoots, indicating their insensitivity to MeJA, while WT and Cl1 H showed susceptibility to MeJA treatment. HSD, honest significant difference; JA, jasmonic acid; MeJA, methyl jasmonate; WT, wild type.

Inhibition of root growth is a well-defined physiological effect of JA and has been used in many forward genetic screens to discover JA-insensitive mutants [8]. To examine whether the phenotypic variations between flowers of WT and transgenic lines are due to JA-insensitivity, the effects of JA on root growth were investigated. While root length of WT and transgenic plants was similar on MeJA-free medium, significant inhibition of root growth was observed for both WT and Cl1 H seedlings on medium containing MeJA (25 μM) (Figure 7a). For Cl2 G and Cl3 A, which express high levels of *PpJAZ1* transgene, less inhibition of roots in MeJA-containing medium was recorded (Figure 7a,b). This effect was even more pronounced with prolonged exposure (40 days) to high concentrations of MeJA (50 μM): although WT and Cl1 H plants barely grew, Cl2 G and Cl3 A plants developed healthy roots and shoots in high-MeJA medium (Figure 7c).

**Figure 8:**
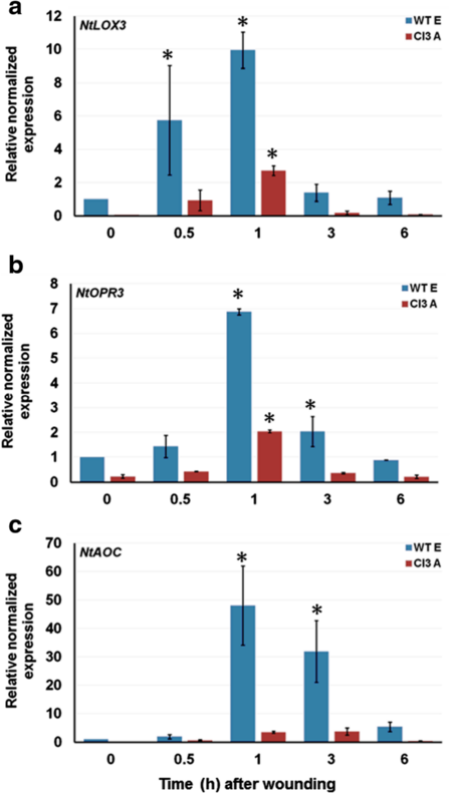
**PpJAZ1 tobacco plants show low transcription levels of JA-biosynthesis genes after mechanical wounding.** Analyses of *Nt-LOX3* **(a)**, *Nt-OPR3* **(b)** and *Nt-AOC* **(c)** gene expression in WT and Cl3 A plants after 0.5, 1, 3 and 6 hours of mechanical wounding. The expression of each gene was normalized to that of *NtActin* in each sample. The results are the mean ± SE of three biological replicates. Values marked with an asterisk (*) are significantly greater than WT control, according to the Tukey-Kramer HSD (*P* <0.05). HSD, honest significant difference; JA, jasmonic acid; SE, standard error; WT, wild type.

Activation of JA-biosynthesis genes in plants has repeatedly been reported in response to herbivorous insect attack [11,34]. Mechanical wounding is mostly used to mimic feeding by herbivores, especially generalists. In order to investigate JA sensitivity in *PpJAZ1* transgenic tobacco plants, the induction kinetics of tobacco *NtLOX3*, *NtOPR3* and *NtAOC* genes, encoding essential enzymes for JA biosynthesis, were monitored in WT and Cl3 A leaves at 0.5, 1, 3 and 6 hours of mechanical wounding. Expression of all genes was significantly lower (*P* <0.001) in untreated-Cl3A plants (0 hours) compared to untreated-WT plants. After wounding, the three genes showed similar induction kinetics in both genotypes, but expression levels were significantly higher in WT compared to Cl3 A (Figure 8). For instance, *NtAOC* transcript abundance increased 41-fold in WT leaves within one hour of wounding, whereas Cl3 A-wounded leaves showed only a four-fold increase in *NtAOC* transcripts, compared to the control (Figure 8c). These results along with the root inhibition assay indicate that PpJAZ1 transgenic tobacco plants show clear features of JA-insensitivity.

### Differential regulation of JA-related genes in leaves and petals

JA biosynthesis genes are regulated by a positive feedback mechanism, meaning that JA itself can induce the expression of its biosynthetic genes [35-37]. The induction of JA biosynthesis genes does not necessarily require *de novo* synthesis of transcriptional activators but rather involves the removal of potential suppressors, that is, JAZ proteins [35]. Although this might explain the significantly low level of JA-biosynthesis genes in wounded and non-wounded Cl3 A, it cannot explain the fertility of these plants which is also a JA-dependent trait. These data had led us to hypothesize that JA biosynthesis downstream of PpJAZ1 might be differentially regulated in leaves and flowers. To examine this notion, the expression of *NtAOC*, *NtLOX3* (13-LOX) and *NtOPR3* were studied in leaves and flower petals of 25 transgenic and three WT tobacco plants. MYC2, ARF8 and MYB21 are known positive regulators for the JA biosynthesis pathway and flower opening [8,38]. Genes encoding these proteins (*NtMYC2a*, *NtMYC2b*, *NtARF8* and *NtMYB21*) were also investigated in WT and transgenic plants. The results of gene expression analyses are presented in Table 1 and Additional file 6: Tables S3, S4 and can be summarized in three main points: 1) the expression of JA biosynthesis genes is asymmetrically associated with their transcription activators. While the expression of *NtAOC* was positively correlated with that of *NtARF8* (r = 0.747 and 0.959) both in leaves and petals, respectively, the expression of *NtLOX3* was highly correlated with that of *NtMYC2a* or *NtMYC2b* (r >0.880) in both leaves and petals. The expression of *NtOPR3* had minimal correlation with any of the studied transcription factors. 2) Although the expression of *NtAOC* was negatively correlated with the level of *PpJAZ1* transgene in leaves (r = -0.564), the expression of these genes was positively correlated in petals (r = 0.728), and so were *NtARF8* and *PpJAZ1*. On the other hand, the expression of *NtLOX3* and its associated genes, *NtMYC2a* and *2b*, was negatively correlated with the expression of the transgene both in leaves and petals. 3) *NtMYB21* did not show any detectable expression in leaves, but was detected in petals. The expression of this gene in petals was negatively correlated with that of *PpJAZ1* (r = -0.715), which is in agreement with the proven role of this gene in flower opening [38-40]. These results strongly support our argument that JA-biosynthesis downstream of PpJAZ1 is divergent in leaves and petals, the implications of which are discussed below.

**Table 1:** **Correlation matrix of transgene-related gene expression**

**Leaves**	*NtAOC*	*NtARF8*	*NtlOX3*	*NtMYB21*	*NtMYC2a*	*NtMYC2b*	*NtOPR3*	*PpJAZ1*
**Flowers**								
*NtAOC*		0.747^**^	-0.043	NA	-0.179	-0.221	0.277	-0.564^**^
*NtARF8*	0.959^**^		-0.087	NA	-0.179	-0.317	-0.077	-0.418^*^
*NtlOX3*	-0.608^**^	-0.577^**^		NA	0.908^**^	0.914^**^	0.538^**^	-0.149
*NtMYB21*	-0.632^**^	-0.410^*^	0.283		NA	NA	NA	NA
*NtMYC2a*	-0.606^**^	-0.610^**^	0.889^**^	0.321		0.968^**^	0.380^*^	-0.064
*NtMYC2b*	-0.628^**^	-0.574^**^	0.942^**^	0.412^*^	0.779^**^		0.438^*^	-0.098
*NtOPR3*	0.412^*^	0.336	-0.311	-0.106	-0.214	-0.268		-0.026
*PpJAZ1*	0.728^**^	0.635^**^	-0.339^*^	-0.715^**^	-0.430^**^	-0.426^*^	0.312	

NA: Not applicable because the expression of *MYB21* was not detected in tobacco leaves. *Values are statistically significant (*P* ≤0.05). **Values are statistically significant (*P* ≤0.01). This correlation matrix illustrates pair wise correlations between the levels of gene expression in leaves and petals. Correlations were calculated using Pearson correlation coefficient and based on data recorded for 25 transgenic and three wild-type tobacco plants.

### JA-insensitivity due to PpJAZ1 stability

**Figure 9:**
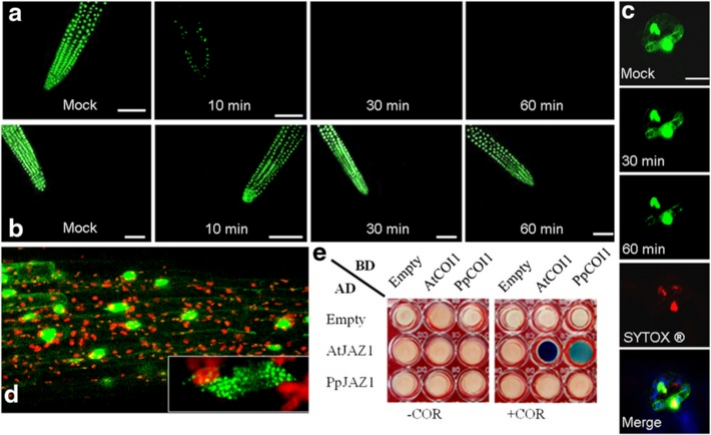
**PpJAZ1 is not subject to JA-induced degradation, and does not interact with PpCOI1. (a-b)** Arabidopsis roots expressing AtJAZ1-GFP **(a)** or PpJAZ1-GFP **(b)** were monitored with confocal laser scanning microscopy after treatment with water (mock) or MeJA (50 μM) for 10, 30 and 60 minutes. **(c)** The stability of PpJAZ1 was also examined in peach leaves transiently expressing PpJAZ1-GFP chimeric protein. Two guard cells showing GFP fluorescence were monitored after 10, 30 and 60 minutes of MeJA (50 μM) treatment (upper three panels). The location of the nucleus is indicated by SYTOX® red staining (fourth panel). The localization of PpJAZ1 in the nucleus was verified with the merged view (bottom panel), scale bars = 10 μm. **(d)** In Arabidopsis roots, PpJAZ1-GFP was present in the nucleus. At the bottom right is a magnified view of the nucleus showing nuclear protein bodies (NPBs) of different sizes. Scale bar = 50 μm. **(e)** Yeast two-hybrid assays revealed no interaction of PpJAZ1 with either PpCOI1 or AtCOI1. No blue colonies were observed in COR-free medium for all assays. In media supplemented with COR (200 μM)), however, only positive controls with AtJAZ1-AtCOI1 proteins showed dark blue colonies. PpCOI1 with AtJAZ1 gave rise to light blue colonies, indicative of lower interaction affinity between the corresponding proteins. JA, jasmonic acid; MeJA methyl jasmonate.

The JA-insensitivity observed in transgenic tobacco plants along with the inability of JA treatment to rescue the CL phenotype in Cl3 A (see Additional file 7: Movie S1), suggests that PpJAZ1 is a highly stable JAZ protein. We confirmed this by investigating *Arabidopsis* roots expressing PpJAZ1-GFP and those expressing AtJAZ1-GFP after 10, 30 and 60 minutes of MeJA (50 μM) treatment. Fluorescence intensity of AtJAZ1-GFP chimeric protein decreased substantially within 10 minutes of MeJA treatment and disappeared altogether after 30 minutes, indicative of complete degradation of AtJAZ1 (Figure 9a), in agreement with previous results [41,42]. By contrast, PpJAZ1-GFP fluorescence remained unchanged after 10, 30 and 60 minutes of MeJA treatment (Figure 9b). The stability of PpJAZ1 was further confirmed in its native cellular environment by transiently expressing PpJAZ1-GFP chimeric protein in peach leaves (Figure 9c). Although PpJAZ1 is localized in the cell nucleus, the close-up view indicates the existence of nuclear protein bodies (NPBs) (Figure 9d). The formation of NPBs has been reported for other signaling molecules as well [43].

The stability of JAZ proteins was demonstrated previously for natural splice-variants that lack the Jas motif or when the Jas motif is purposely removed from the complete protein [44,45]. In both cases, the absence of the Jas domain hindered the interaction of JAZ proteins with COI1, which targets these proteins for degradation via the ubiquitin/26S proteasome pathway. The interaction between JAZ proteins and COI1 is facilitated by JA- isoleucine (JA-Ile) [15,21], the most bioactive JA, or coronatine (COR), which is structurally similar to (+)-7-iso-JA-lle [46]. To examine whether the stability of PpJAZ1 was due to lack of interaction with COI1, we used a yeast two-hybrid assay. Although the positive control represented by AtJAZ-AtCOI1 showed interaction in the presence of COR (200 μM), PpJAZ1 did not show interaction with AtCOI1 or PpCOI1 either in the absence or presence of COR (Figure 9e). Together, these results suggest that the inability of PpJAZ1 to interact with PpCOI1 leads to its heightened stability.

## Discussion

### The role of JA during peach flower development

The role of JA as a floral display regulator in peach was initially supported by the significantly higher levels of JA and JA-Ile in petals of pCL peach variety ‘Glowing Star’ compared to the CH variety ‘VABM29’. Similarly, a recent report [32] indicated that JA levels in the corollas of *N. attenuata* flowers peak at two DAP (days after protrusion) and then decrease sharply until anthesis [32], suggesting that sustained JA concentration might be associated with restricted petal growth as is the case with the non-showy peach varieties. Indeed, the negative impact of JA on petal elongation has already been demonstrated in *Arabidopsis* JA-deficient mutant *opr3* which exhibits enhanced petal growth compared to WT flowers [47]. Furthermore, JA-deficient *N. attenuata* mutants (*ir-aoc*) produced corollas that are longer than those of the WT, but their flowers failed to open [32]. In the latter example, the application of COR to young flower buds (≤9 mm) encouraged flower opening, but COR failed to rescue flower opening when it was added to more developed buds. Collectively, these observations indicate that JA transiently accumulates in patterns regulated by several feedback loops during the transition from bud stage to anthesis.

The uncoupled induction of JA-biosynthesis genes and the increase in JA levels in the petals of the pCL peach variety ‘Glowing Star’ during anthesis suggests that *de novo* synthesis is not the case, but it may be rather the transfer of JA to the petals from adjacent organs. Indeed, JA biosynthesis is thought to occur in stamen filaments, and JA can be transported to petals by diffusion or through water flow [48]. The early anther dehiscence of peach pCL flowers (Figure 1d), the enhancement of anther dehiscence by MeJA treatment (Figure 2e), the small petal surface area of peach pCL flowers and the negative effect of MeJA application on the petal size (Figure 2d), all suggest that JA is transported from stamens to petals shortly after anther dehiscence, leading to cessation of petal growth.

Such coordination between stamens and petals through JA is crucial for the synchronization among anther dehiscence, petal expansion and flower opening, which enables cross-pollination in CH flowers [13]. Further support for this hypothesis is provided by *Arabidopsis* JA-biosynthesis (that is, *aos-2*) and perception (that is, *coi1-1*) mutants, which show defects in anther development while producing large petals [38]. In these mutants, petal growth is delayed compared to the WT, but petals continue to grow after pollination and subsequently become larger than WT petals. Similarly, mutations in *OsJAR1*, the JA-amino acid synthetase, resulted in rice plants with defective anther dehiscence and flowers that stayed open for several days [20], implying that the signal mediated through JA to regulate petal growth and flower opening is absent in these mutants. On the contrary, this signal appears to occur early in the pCL peach flowers, where anther dehiscence takes place early and, hence, the petals grow only slightly at the late developmental stages, avoiding an inefficient allocation of resources to floral display in this autogamous species (Figure 1c). It has been shown already that the peach stigma naturally matures well before the ovary [49], which is also consistent with cleistogamy.

### PpJAZ1-mediated cleistogamy

Among the nine JAZ genes investigated, only *PpJAZ1* showed different induction kinetics before and after flower opening. The higher induction of *PpJAZ1* in the CL flowers at stage V, along with the existence of potential binding sites for transcription factors with crucial roles in flower growth and development in its promoter region, strongly suggest that *PpJAZ1* is associated with the diverse phenotypes of peach flowers. The ectopic expression of *PpJAZ1* in tobacco was coupled with ceased flower opening and reduced corolla coloration, which further confirm the potential regulatory role of *PpJAZ1* during late stages of flower development. Accumulation of anthocyanin, the pigment which gives tobacco flowers their color [50], is induced by JA. The expression of anthocyanin biosynthetic genes *DFR*, *LDOX*, and *UF3GT* is almost abolished in *Arabidopsis coi1* plants [51], which points to a molecular link between JA signaling and anthocyanin accumulation. Hence, the loss of pigmentation in *PpJAZ1* tobacco flowers likely represents a form of JA-insensitivity in these plants.

The closed but self-fertilized CL phenotype mediated by the ectopic expression of *PpJAZ1* in tobacco is reminiscent of knocking-down *EOBII* or its homologs in petunia (*Petunia x hybrid*), coyote tobacco (*N. attenuata*), or ornamental tobacco (*N. langsdorffii x N. sanderae*) through the RNAi-based approach [39,52]. Similarly, knocking-out *EOBII* homologs in *Arabidopsis* (*AtMYB21* and *AtMYB24*) through T-DNA insertion [40] resulted in a CL flower phenotype. In case of *Arabidopsis* and ornamental tobacco, such defects in flower opening were incidental observations and not the focus of experimentation. Interestingly, the expression level of *EOBII* homolog in tobacco (*NtMYB21*) was negatively correlated with the level of *PpJAZ1* transgene expression in these plants (Table 1 and Additional file 6: Table S4), indicating that flower closure in these plants might be mediated through the inhibitory effects of *PpJAZ1* over the transcription of *NtMYB21*. It has also been demonstrated that JAZ proteins can physically interact with members of R2R3 MYB transcription factors (that is, MYB75 and MYB21) and, hence, attenuate JA-mediated anthocyanin accumulation and JA-mediated anther development, respectively [24,25]. Indeed, tobacco transgenic lines with high transcript levels of *PpJAZ1* (that is, Cl3 O, Cl3 Q, Cl2 J) had short stamen filaments (see Additional file 6: Table S4) and showed low corolla pigmentation (Figure 5a). Although the application of GA with sucrose and *t*-cinnamic acid partially restored flower opening in *ir-PhEOB11* petunia plants [39], neither GA nor MeJA treatments (50 μM and up to 500 μM) could restore flower opening in cleistogamous Cl3 A flowers (see Additional file 7: Movie S1). Given the demonstrated stability of PpJAZ1, these results together suggest that PpJAZ1 might have arrested the activity of MYB75, MYB21 or other MYB transcription factors which regulate the chasmogamous nature of WT tobacco flowers.

### PpJAZ1 stability

Ectopic expression of *PpJAZ1* in tobacco led to JA-insensitivity similar to that obtained after expressing stabilized versions of *Arabidopsis* JAZ1, JAZ3 or JAZ10, which lack Jas motifs (JAZΔJas), except that transgenic *PpJAZ1* plants are generally fertile. Mutations in, or the removal of, Jas motif lead to JAZ stabilizations and consequent JA-insensitivity [21,53,54]. Although the half-life of AtJAZ1 in the presence of JA was determined to be 1.4 minutes [42], PpJAZ1-GFP protein expressed in *Arabidopsis* roots or peach leaves did not show any degradation even after 60 minutes of MeJA treatment. Yeast two-hybrid assays further confirmed the stability of PpJAZ1, where no interactions were detected between PpJAZ1 with either PpCOI1 or AtCOI1. The comparison among PpJAZ1 and all 12 *Arabidopsis* JAZs did not show any differences in Jas motif or degron sequence that would abolish PpJAZ1 ability to interact with COI1 and accordingly its degradability after MeJA treatment. The only difference in PpJAZ1 was the Asn 246, which does not exist in *Arabidopsis* JAZs (see Additional file 3: Text S1). How this difference might affect PpJAZ1 interaction with COI1 is yet to be investigated.

### PpJAZ1 regulates spatial localization of JA signaling

Stability of JAZ proteins has recently been attributed to the spatial localization of JA signaling in different organs as well as the JA-mediated resource allocation trade-offs. For instance, recent reports have demonstrated that JA pathway regulation differs between roots and aerial parts due to NINJA (Novel Interactor of JAZ), which along with other elements constitutes a repressor complex negatively regulating JA-signaling. Results showed that NINJA are active mainly in roots in order to repress JA-mediated root inhibition and allow normal root growth. This role mediated by NINJA is thought to involve stable JAZ proteins which make up a part of the repressor complex [55]. Furthermore, plants can manage resource allocation in aerial parts through enhancing the stabilization of JAZ proteins under non-optimum light conditions, which in turn encourages growth more than defense [56]. Similarly, the differential regulation of JA-related genes in leaves and flowers of *PpJAZ1* transgenic tobacco plants suggests PpJAZ1 as a molecular switch to JA signaling in these organs. One of the JA signaling branches operates in leaves upon wounding/herbivore attack and probably involves JAZ-MYC interactions [26,57] and their downstream genes, for example, *LOX3*, whereas another branch operates during floral development to regulate aspects of male fertility and flower opening and is likely controlled by JAZ-MYB interactions [24,25] and the up-regulation of other JA-biosynthesis genes, for example, *AOC* through ARF6/8 (Table 1).

From an ecological perspective, such a regulatory role of PpJAZ1 should enhance plant fitness in harsh environments and preserve their ability to reproduce. Harsh environmental conditions encourage the production of cleistogamous flowers [5]. Hence, the higher expression of *PpJAZ1* transcripts in the pCL non-showy peach flowers could be evolutionarily driven by stressful conditions that favor induction of these stable forms of JAZ proteins in order to switch resources toward self-pollination. Among the features associated with enhancing selfing is the reduction in floral display features (that is, showy appearance) that serve to attract pollinators. Interestingly, earlier research indicated that cleistogamy protects peach flowers from both extreme weather [58] and diseases, such as flower blight caused by *Monilinia sp*., which usually attacks flowers through stamen filaments [59]. Further investigations using different JA-deficient mutants could shed more light on these potential associated benefits.

## Conclusions

Our results clearly show the involvement of PpJAZ1, a stable form of a key intermediate of the JA signaling cascade, in regulating the variations of floral display and self-pollination. Using tobacco as a heterologous experimental system we demonstrated that *PpJAZ1* expression can convert chasmogamous flowers into cleistogamous flowers. Our results also point toward a role for PpJAZ1 as a molecular switch for JA signaling in leaves and flowers, a potential mechanism by which plants shift to selfing under harsh environmental conditions. This represents a previously uncharacterized role of JAZ proteins in plants.

## Methods

### Measurements of floral organ dimensions

Petal surface area (cm^2^), stamen length (cm), pistil length (cm) and anther dehiscence (%) were recorded for 10 peach flowers each in different stages (II, III, IV and V) of development. Petal surface area was calculated by measuring the height and width of each petal using a ruler and considering the mostly oval shape of petals, the surface area was calculated as, (height × width) × 0.8. The percentage of dehiscent anthers was recorded by dividing the number of visibly dehiscent anthers to the total number of anthers and multiplying by 100.

### Quantification of JA and JA-Ile

Petal tissues collected from ‘Glowing Star’ and ‘VAMB29’ were frozen in liquid nitrogen, lyophilized and stored at -20°C. Extraction and quantification of JA and JA-Ile was performed as described previously [60]. Briefly, 80% methanol extracts from three biological replicates were subjected to LC-ESI-MS/MS using Agilent G4790A equipped with a ZORBAX Eclipse Plus C18 column (1.8 μm, 2.1 × 50 mm).

### Hormone treatments and mechanical wounding

The effect of JA on peach flower opening was examined in ‘Glowing Star’ by selecting branches (approximately 75 cm height and 1 cm thickness) carrying stage II flowers. Five branches were sprayed with a solution containing 0.01% Triton X-100 mixed with either MeJA (100 μM) or water. Both MeJA- and water-treated branches were placed vertically in pots containing distilled water and stored at room temperature. After 24, 48 and 72 hours, 12 flowers were randomly collected from each treatment to record the petal surface area and percentage of anther dehiscence, as described above.

For the wounding experiment in tobacco, leaves from WT E and Cl3 A were punctured several times by a needle and then crushed with a hemostat. Wounded leaves were detached from the plant after 0.5, 1, 3 and 6 hours, frozen immediately in liquid nitrogen and then stored at -80°C for RNA extraction. Intact tobacco leaves were collected at 0 hours to serve as controls.

### Cloning and in silico analysis

The partial sequences of *PpOPR3*, *PpLOX3* and *PpActin* were isolated from a peach cDNA library generated from ‘VABM29’ peach leaves using Reverse Transcription Polymerase Chain Reaction (RT-PCR). Primers to amplify these genes were designed according to the sequences available at the peach EST database [61]. Six JAZ genes (*PpJAZ1, PpJAZ3, PpJAZ4, PpJAZ5, PpJAZ7* and *PpJAZ11*) were identified based on the assembly of JAZ sequences using the CAP3 Sequence Assembly Program [62]. The full-length nucleotide sequences of these genes were further isolated from cDNA libraries generated from ‘VAMB29’ leaves, using RT-PCR. The resulting PCR fragments were cloned into pGEM-T easy vector (Promega, Madison, WI, USA), sequenced and compared with database sequences using the BLAST program. Three PCR fragments with unexpected lengths were obtained from the previous approach and proved to contain a Jas domain. The full-length nucleotide sequences of these fragments were acquired using 3’- and 5’- RACE kits (Invitrogen, Burlington, ON, Canada) according to the manufacturer’s instructions. The PCR fragments were cloned, sequenced and subsequently designated *PpJAZ8*, *PpJAZ9* and *PpJAZ10* based on shared similarities with other JAZ orthologs in GenBank. A partial sequence of *PpCOI1* was obtained from the peach EST library using BLASTp against *ArabidopsisCOI1* (*AtCOI1*). The full-length nucleotide sequence of *PpCOI1* was isolated from the peach cDNA library generated from ‘VABM29’ leaves, using the same approach described above. Multiple comparisons of *P. persica* JAZ (PpJAZ) proteins with orthologs from different plant species were performed using ClustalX [63]) and GeneDoc [64]). The phylogenetic tree was generated using MEGA 5.05 [65], applying the maximum likelihood bootstrap method (1,000 replicates). An initial draft of the assembled peach genome was recently released and, therefore, the sequences for *JAZs* and other genes investigated in this study can be retrieved directly from Phytozome [66] using the BLAST tool or can be accessed through the National Center for Biotechnology Information (NCBI) database using accession numbers (see Additional file 6: Table S6). Sequences for *NtMYB21* and *NtARF8* were obtained by Blasting TOBFAC database [67] against the corresponding *Arabidopsis* sequences of these genes (accession # AT3G27810 and ACB30882), respectively. Other tobacco sequences were obtained either by direct cloning from the tobacco cDNA library (that is, *NtAOC*, *NtOPR3* and *NtLOX3*) or directly retrieved from the NCBI database (that is, *NtMYC2a* and *NtMYC2b*).

### Isolation and analysis of PpJAZ1 promoter sequence

Genomic DNA was extracted from ‘VABM29’, ‘Bounty’, ‘Glowing Star’ and ‘V85331’ leaves using a Plant/Fungi DNA Isolation Kit (Norgen, Thorold, ON, Canada). The 5’ upstream region of *PpJAZ1* was isolated using the Universal Genome Walker Kit (Clontech, Palo Alto, CA, USA). Nested PCR reactions were performed using reverse primers in the coding region (see Additional file 6: Table S5) and adaptor primers provided by the manufacturer. PCR-amplified fragments were cloned and sequenced as explained above. Promoter sequences were analyzed using the Plant Promoter Analysis Navigator (PlantPAN) [68].

### RNA isolation and transcription analyses

Total RNA was extracted from peach and tobacco flowers using the CTAB method [69], and from tobacco leaves using RNeasy Plant Mini Kit (Qiagen, Toronto, ON, Canada). The first-strand cDNA was synthesized using 2.5 μg of DNase treated total RNA and the SuperScript® VILO™ cDNA Synthesis Kit (Invitrogen, Burlington, ON, Canada) in a total volume of 20 μl. Quantitative real time-PCR (qRT-PCR) was performed using the CFX connect real-time Detection System (Bio-Rad, Mississauga, ON, Canada) and SsoFast™ EvaGreen® Supermix (Bio-Rad). The primers used to detect transcript levels of peach and tobacco genes are listed (see Additional file 1: Table S5). Specific amplification of target genes was further verified using a dissociation curve program from 65°C to 95°C. The cycle number at which the fluorescence passed the threshold (CT) for all selected genes was normalized to the CT value of β-actin gene whose expression remained constant among various stages and treatments (see Additional file 8: Figure S4). Three different approaches for calculating gene expression were used based on the purpose of the experiment. Relative gene expression was used when the control (the reference) sample cannot be determined. In this approach, the gene expression is calculated as 2^-∆CT^, where the ∆CT represents the CT of the gene of interest minus the CT of β-actin. The relative normalized expression approach was used when the control sample is known. This method is based on the 2^-∆∆CT^, where ∆∆CT represents the ∆CT of the treatment minus the ∆CT of the control sample. Lastly, the gene regulation approach is the same as the relative normalized expression approach except a plus or minus sign is added to the value in order to show the up-regulation or down-regulation of the gene, respectively, compared to the control. Results were statistically analyzed using the CFX manager software (Bio-Rad). The primers used to detect transcript levels of peach and tobacco genes are listed (see Additional file 6: Table S5).

### Generation of transgenic plants

*PpJAZ1* full-length cDNA was PCR amplified using the primers *PpJAZ1.F* and *PpJAZ1.R* (see Additional file 6: Table S5) and cDNAs generated from ‘Glowing Star’ flowers. The amplified fragment was digested with *BamHI* and cloned into the pGreen binary vector [70], in-frame with the N-terminal of GFP and upstream of the CaMV dual 35S promoter. The PpJAZ1-GFP construct was introduced into *Agrobacterium tumefaciens* C58 (harboring pSoup plasmid) by electroporation. Tobacco (*Nicotiana tabacum* L. cv. PetH4) plants grown in GA-7 culture vessels (Magenta®, Chicago, IL, USA) were transformed using the leaf-disc method as described previously [71]. The transformation of *Arabidopsis* (Columbia 0) plants was performed using the floral dip method [72].

### MeJA effect on seedling growth

Seeds of WT E, Cl1 H, Cl2 G and Cl3 A plants were germinated on MS medium with or without MeJA (25 μM). Germination plates were placed at 4°C for four days to break seed dormancy and then placed vertically in growth rooms for 12 days at 25°C. Two plates from each genotype were used to measure root lengths and the whole experiment was conducted in triplicate. The effect of long-term exposure to a high concentration of MeJA was tested by growing the seeds for 40 days in glass tubes containing MS medium with MeJA (50 μM).

### *In vivo* degradation of JAZ-GFP fusion protein

JA-mediated PpJAZ1 degradation was tested in *Arabidopsis* roots and peach leaves. *Arabidopsis* roots were collected from T3 transgenic plants grown in MS medium with 3% sucrose. The roots were observed initially for GFP fluorescence and then rinsed in MeJA (25 μM) for 10, 30 and 60 minutes. After each stage, root samples were mounted on microscope slides and observed using a Leica TCS-SP5 with a 488 nm Ar laser and a 543 nm HeNe laser (Leica) and using a Leica DM6000B microscope equipped with a 40 X water immersion lens. *Arabidopsis* seedlings expressing At-JAZ1-GFP chimeric protein were subjected to the same treatments mentioned above as positive controls.

To investigate the stability of the PpJAZ1-GFP chimeric protein in peach, mature peach leaves were bombarded with 10 μl (1 μg/μl) PpJAZ1-GFP plasmid using a Pds 1000/He biolistic device (Bio-Rad) as per the particle bombardment protocol [73].

### Yeast-two hybrid (Y2H) assay

The full-length cDNAs encoding PpJAZ1, PpCOI1, AtJAZ1 and AtCOI1 were PCR amplified using RT-PCR with the primers listed (see Additional file 6: Table S5). Y2H assays were performed with the Matchmaker Gold Yeast two-hybrid System (Clontech, Mountain View, CA, USA).

### Statistical analysis

One- and two-way analysis of variance (ANOVA) was performed using the ANOVA, GLM or MIXED procedures of SAS statistical software (release 9; SAS Institute, Cary, NC, USA). The Tukey-Kramer HSD test was used to compare means. All parameters were tested for normality prior to analysis of variance, and a *log* transformation was performed when required. Treatment means and standard errors presented in figures were calculated from non-transformed data.

## Additional files

Additional file 1: Figure S1. Difference between showy and non-showy appearance in peach is attributed to cell expansion. Micrographs showing cell length and cell width (μm) in petals of VABM29 (a) and V85331 (b) flowers taken at the anthesis stage. Scale bar = 40 μm. Length and width of petals cells in VABM29 (*n* = 25) and V85331 (*n* = 25) (c). Additional file 2: Figure S2. Expression of JA biosynthesis genes in peach flowers. The expression of two JA-biosynthesis genes, *PpLOX3* (a) and *PpOPR3* (b) was quantified in flowers of showy and non-showy varieties at 0% (Stage II) and at 100% anther dehiscence (Stage V). The expression of each gene was normalized to that of *Ppactin*. Values are the mean and standard error of three biological replicates. Means in each column having the same letter are not significantly different (*P* <0.05, Tukey-Kramer HSD test). Additional file 3:
**Text S1.** Multiple sequence alignments identified seven distinct classes of peach JAZ proteins. Deduced amino acid sequences of JAZ proteins from peach and other plant species were aligned using Clustal X and edited using GeneDoc. The two characteristic motifs, ZIM and Jas, were identified for all JAZ proteins and are indicated by red and green solid lines, respectively. A weakly conserved N-terminal (NT) motif was identified in Class I -JAZs and indicated by a blue solid line. A highly conserved motif was identified in Class II -JAZs and indicated by stars. An EAR-like element was identified at the N-terminus of Class III - JAZs and is indicated by red letters. Additional file 4: Figure S3. Phylogenetic analysis of peach JAZ proteins. Phylogenetic tree was constructed based on the available sequences for *Arabidopsis thaliana* (*At*), *Nicotiana attenuata* (*Na*), *Solanum lycopersicum* (*Sl*), *Vitis rupestris* (*Vr*), *Nicotine tabacum* (*Nt*) along with *Prunus persica* (*Pp*) JAZ proteins. PpJAZ proteins are indicated by black circles in the phylogram. Additional file 5:
**Text S2.** PpJAZ1 promoter sequences isolated from different peach varieties. The 1,226 bp sequence upstream the translational start site of *PpJAZ1* was isolated from the genomic library of ‘VABM29’, ‘Bounty’, ‘Glowing Star’ and ‘V85331’ peach varieties. Alignment of promoter sequences using Clustal X identified six SNPs among peach varieties. Further analysis of promoter sequences using PlantPAN web tool showed that one of these SNPs is located within the binding site of AINTEGUMENTA transcription factor as indicated by the red stars. Additional file 6: Table S1.
*cis*-regulatory elements identified in *PpJAZ1* promoter sequence. **Table S2.** Phenotypic characterization of *PpJAZ1* tobacco lines. **Table S3.** Differential gene expression in leaves of transgenic tobacco plants. **Table S4.** Differential gene expression in petals of transgenic tobacco plants. **Table S5.** Sequences of the oligonucleotides described in Methods. **Table S6.** Accession numbers for genes used in this study. Additional file 7:
**Movie S1.** Treatment of transgenic plants with MeJA did not restore normal flower opening. Flowers of Cl3 which represent typical cleistogamous flowers did not show any flower opening after 24 hours of treatment with MeJA (100 μM). Additional file 8: Figure S4. The uniformity of *β-Actin* expression in different samples. The qRT-PCR amplification plot for *PpActin*, *PpJAZ5* (a), *NtActin* and *NtMYB21* (b) in cDNAs generated from peach and tobacco flowers, respectively. The X-axis shows the number of PCR cycles and the Y-axis shows the relative fluorescent units (RFU). The amplification plot was generated for six cDNA samples and three technical replicates for each sample.

## Abbreviations

ABA: abscisic acid
AOC: allene oxide cyclase
AOS: allene oxide synthase
bp: base pair
CH: chasmogamous
CL: cleistogamous
COI1: coronatine insensitive 1
EOBII: emission of benzenoids II
GA: gibberellic acid
GFP: green fluorescent protein
HSD: honest significant difference
JA: jasmonic acid
JA-Ile: JA-isoleucine
JAZ: jasmonate ZIM-Domain
LOX3: lipoxygenase 3
MeJA: methyl jasmonate
NPBs: nuclear protein bodies
OPR3: OPDA reductase 3
pCL: pre-anthesis cleistogamous
SNP: single nucleotide polymorphism
WT: wile type
