# Supplementary material for: A stable JAZ protein from peach mediates the transition from outcrossing to self-pollination
Source: BMC Biol. 2015 Feb 13;13:11. doi: 10.1186/s12915-015-0124-6 (PMC4364584; doi:10.1186/s12915-015-0124-6)
Supplement: Additional file 1: Figure S1. — Difference between showy and non-showy appearance in peach is attributed to cell expansion. Micrographs showing cell length and cell width (μm) in petals of VABM29 (a) and V85331 (b) flowers taken at the anthesis stage. Scale bar = 40 μm. Length and width of petals cells in VABM29 (n = 25) and V85331 (n = 25) (c). [file 12915_2015_124_MOESM1_ESM.pdf]

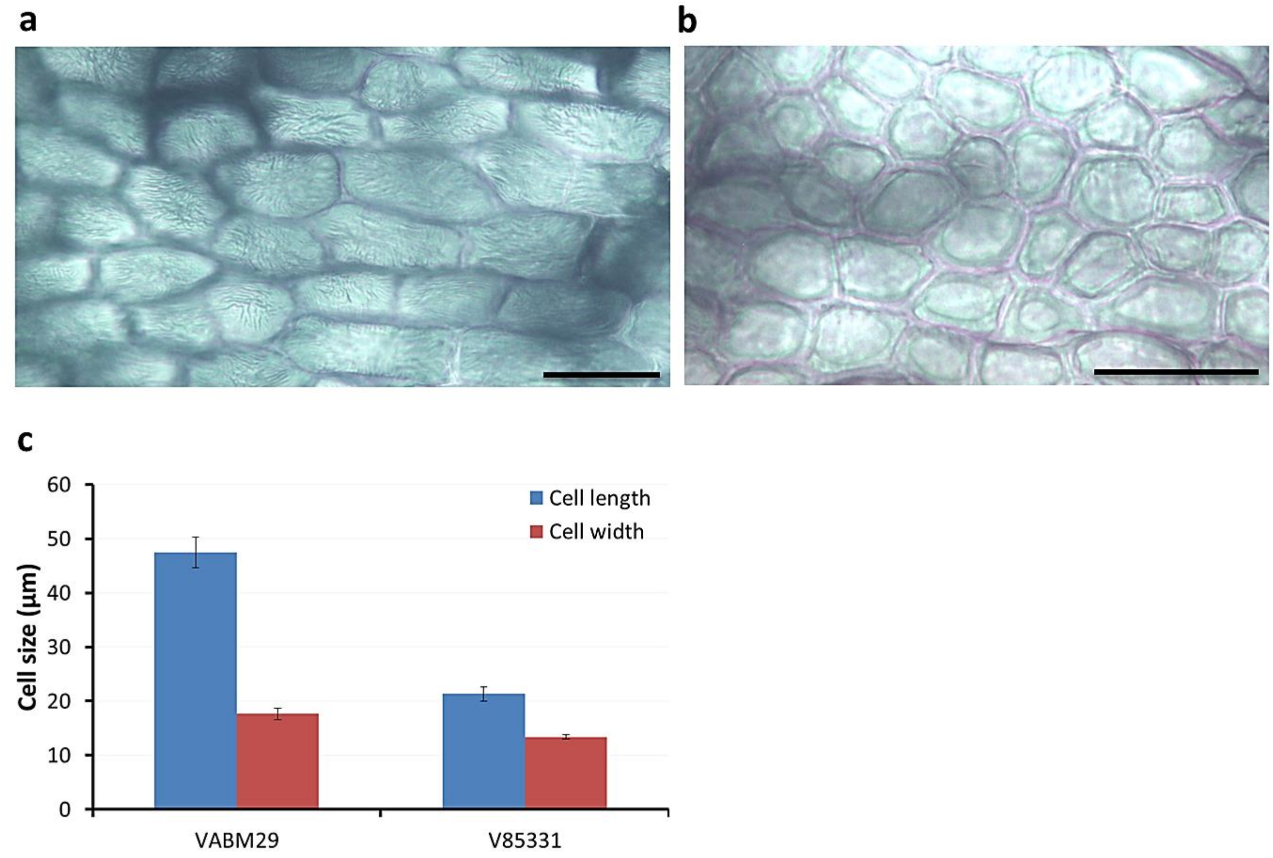

Figure S1: Showy and non-showy appearance in peach is attributed to cell size. Micrographs showing cell length and cell width (µm) in petals of VABM29 (a) and V85331 (b) flowers taken at the anthesis stage. Scale bar = 40 µm. Length and width of petals cells in VABM29 (*n* = 25) and V85331 (*n* = 25) (c).
