# Supplementary material for: A stable JAZ protein from peach mediates the transition from outcrossing to self-pollination
Source: BMC Biol. 2015 Feb 13;13:11. doi: 10.1186/s12915-015-0124-6 (PMC4364584; doi:10.1186/s12915-015-0124-6)
Supplement: Additional file 2: Figure S2. — Expression of JA biosynthesis genes in peach flowers. The expression of two JA-biosynthesis genes, PpLOX3 (a) and PpOPR3 (b) was quantified in flowers of showy and non-showy varieties at 0% (Stage II) and at 100% anther dehiscence (Stage V). The expression of each gene was normalized to that of Ppactin. Values are the mean and standard error of three biological replicates. Means in each column having the same letter are not significantly different (P <0.05, Tukey-Kramer HSD test). [file 12915_2015_124_MOESM2_ESM.pdf]

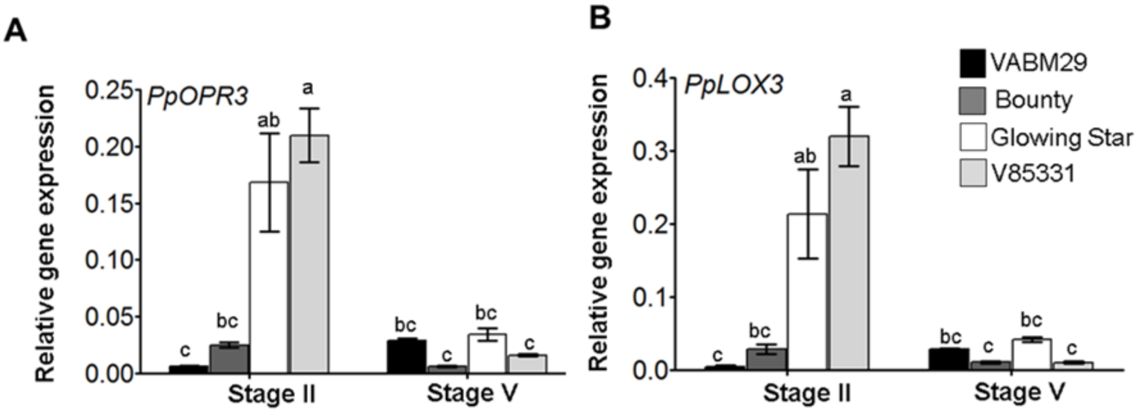

Figure S2: Expression of JA biosynthesis genes in peach flowers. The expression of two JA-biosynthesis genes, *PpLOX3* (a) and *PpOPR3* (b) were quantified in flowers of showy and non-showy varieties at 0% (Stage II) and at 100 % anther dehiscence (Stage V). The expression of each gene was normalized to that of *Ppactin*. Values are the mean and standard error of three biological replicates. Means in each column having the same letter are not significantly different ($P < 0.05$, Tukey-Kramer HSD test).
