# Supplementary material for: A stable JAZ protein from peach mediates the transition from outcrossing to self-pollination
Source: BMC Biol. 2015 Feb 13;13:11. doi: 10.1186/s12915-015-0124-6 (PMC4364584; doi:10.1186/s12915-015-0124-6)
Supplement: Additional file 3: — Text S1. Multiple sequence alignments identified seven distinct classes of peach JAZ proteins. Deduced amino acid sequences of JAZ proteins from peach and other plant species were aligned using Clustal X and edited using GeneDoc. The two characteristic motifs, ZIM and Jas, were identified for all JAZ proteins and are indicated by red and green solid lines, respectively. A weakly conserved N-terminal (NT) motif was identified in Class I -JAZs and indicated by a blue solid line. A highly conserved motif was identified in Class II -JAZs and indicated by stars. An EAR-like element was identified at the N-terminus of Class III - JAZs and is indicated by red letters. [file 12915_2015_124_MOESM3_ESM.pdf]

"Class I"

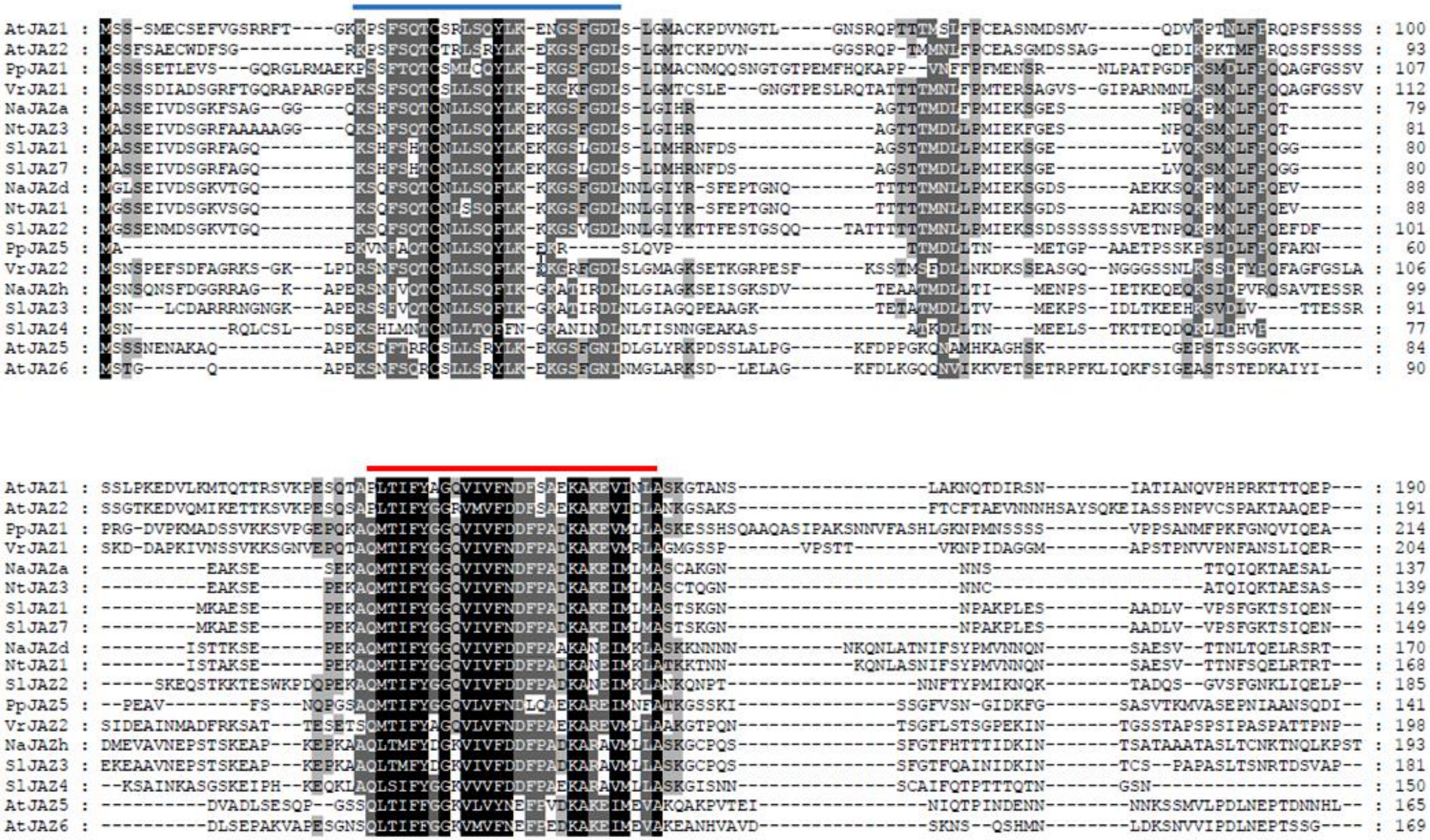
AtJAZ1 : 100
AtJAZ2 : 93
PpJAZ1 : 107
VrJAZ1 : 112
NaJAZa : 79
NtJAZ3 : 81
SlJAZ1 : 80
SlJAZ7 : 80
NaJAZd : 88
NtJAZ1 : 88
SlJAZ2 : 101
PpJAZ5 : 60
VrJAZ2 : 106
NaJAZh : 99
SlJAZ3 : 91
SlJAZ4 : 77
AtJAZ5 : 84
AtJAZ6 : 90
AtJAZ1 : 190
AtJAZ2 : 191
PpJAZ1 : 214
VrJAZ1 : 204
NaJAZa : 137
NtJAZ3 : 139
SlJAZ1 : 149
SlJAZ7 : 149
NaJAZd : 170
NtJAZ1 : 168
SlJAZ2 : 185
PpJAZ5 : 141
VrJAZ2 : 198
NaJAZh : 193
SlJAZ3 : 181
SlJAZ4 : 150
AtJAZ5 : 165
AtJAZ6 : 169

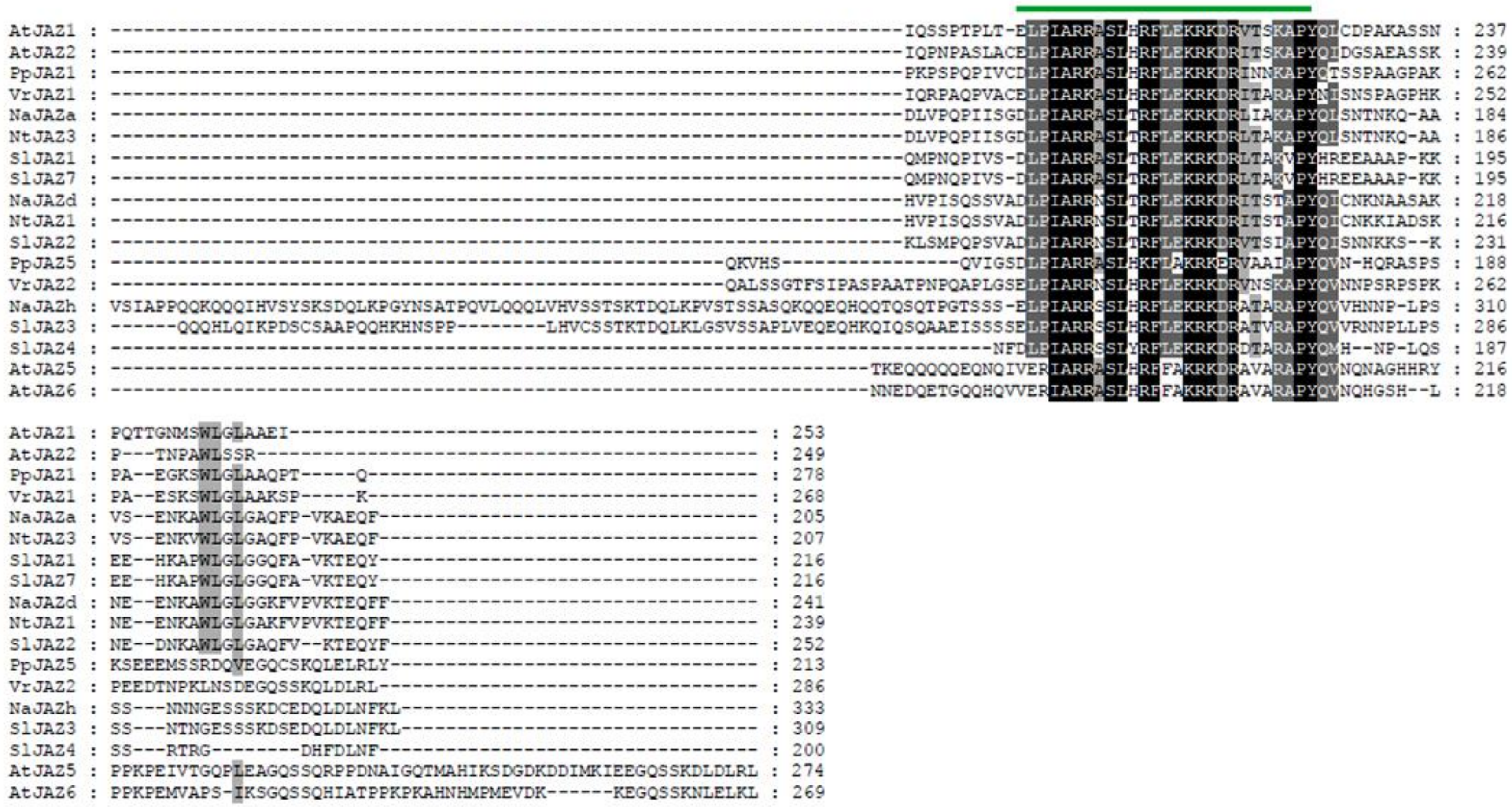

AtJAZ1 : ---IQSSPTPLT-ELPIARRASLHRFLEKRKDRVTSKAPYQICDPAKASSN : 237
AtJAZ2 : ---IQPNPASLACELPIARRASLHRFLEKRKDRITSKAPYQIDGSAEASSK : 239
PpJAZ1 : ---PKPSPQPIVCDLPIARKASLHRFLEKRKDRINNKAPYQTSSPAAGPAK : 262
VrJAZ1 : ---IQRPAQPVACELPIARKASLHRFLEKRKDRITARAPYNISNSPAGPHK : 252
NaJAZa : ---DLVPQPIISGDLPIARRASLTRFLEKRKDRLIAKAPYQISNTNKQ-AA : 184
NtJAZ3 : ---DLVPQPIISGDLPIARRASLTRFLEKRKDRLTAKAPYQISNTNKQ-AA : 186
SlJAZ1 : ---QMPNQPIVS-DLPIARRASLTRFLEKRKDRLTAKVPYHREEAAAP-KK : 195
SlJAZ7 : ---QMPNQPIVS-DLPIARRASLTRFLEKRKDRLTAKVPYHREEAAAP-KK : 195
NaJAZd : ---HVPISQSSVADLPIARRNSLTRFLEKRKDRITSTAPYQICNKNAASAK : 218
NtJAZ1 : ---HVPISQSSVADLPIARRNSLTRFLEKRKDRITSTAPYQICNKKIADSK : 216
SlJAZ2 : ---KLSMPQPSVADLPIARRNSLTRFLEKRKDRVTSIAPYQISNNKKS--K : 231
PpJAZ5 : ---QKVHS---QVIGSDLPIARRASLHKFLAKRKERVAAIAPYQVN-HQRASPS : 188
VrJAZ2 : ---QALSSGTFSIPASPAATPNPQAPLGSELPIARRNSLHRFLEKRKDRVNSKAPYQVNNPSRPSPK : 262
NaJAZh : VSIAPPQQKQQQIHVSYSKSDQLKPGYNSATPQVLQQQLVHVSSTSKTDQLKPVSTSSASQKQQEQHQQTQSQTPGTSSS-ELPIARRSSLHRFLEKRKDRATARAPYQVVHNNP-LPS : 310
SlJAZ3 : ------QQQHLQIKPDSCSAAPQQHKHNSPP--------LHVCSSTKTDQLKLGSVSSAPLVEQEQHKQIQSQAAEISSSSELPIARRSSLHRFLEKRKDRATVRAPYQVVRNNPLLPS : 286
SlJAZ4 : ---NFDLPIARRSSLYRFLEKRKDRDTARAPYQMH--NP-LQS : 187
AtJAZ5 : ---TKEQQQQQEQNQIVERIARRASLHRFFAKRKDRAVARAPYQVNQNAGHHRY : 216
AtJAZ6 : ---NNEDQETGQQHQVVERIARRASLHRFFAKRKDRAVARAPYQVNQHGSH--L : 218
AtJAZ1 : PQTTGNMSWLGLAAEI--- : 253
AtJAZ2 : P---TNPAWLSSR--- : 249
PpJAZ1 : PA--EGKSWLGLAAQPT-----Q--- : 278
VrJAZ1 : PA--ESKSWLGLAAKSP-----K--- : 268
NaJAZa : VS--ENKAWLGLGAQFP-VKAEQF--- : 205
NtJAZ3 : VS--ENKVWLGLGAQFP-VKAEQF--- : 207
SlJAZ1 : EE--HKAPWLGLGGQFA-VKTEQY--- : 216
SlJAZ7 : EE--HKAPWLGLGGQFA-VKTEQY--- : 216
NaJAZd : NE--ENKAWLGLGGKFVPVKTEQFF--- : 241
NtJAZ1 : NE--ENKAWLGLGAKFVPVKTEQFF--- : 239
SlJAZ2 : NE--DNKAWLGLGAQFV--KTEQYF--- : 252
PpJAZ5 : KSEEEMSSRDQVEGQCSKQLELRLY--- : 213
VrJAZ2 : PEEDTNPKLNSDEGQSSKQLDLRL--- : 286
NaJAZh : SS---NNNGESSSKDCEDQLDLNFKL--- : 333
SlJAZ3 : SS---NTNGESSSKDSEDQLDLNFKL--- : 309
SlJAZ4 : SS---RTRG--------DHFDLNF--- : 200
AtJAZ5 : PPKPEIVTGQPLEAGQSSQRPPDNAIGQTMAHIKSDGDKDDIMKIEEGQSSKDLDLRL : 274
AtJAZ6 : PPKPEMVAPS-IKSGQSSQHIATPPKPKAHNHMPMEVDK------KEGQSSKNLELKL : 269

“Class II”

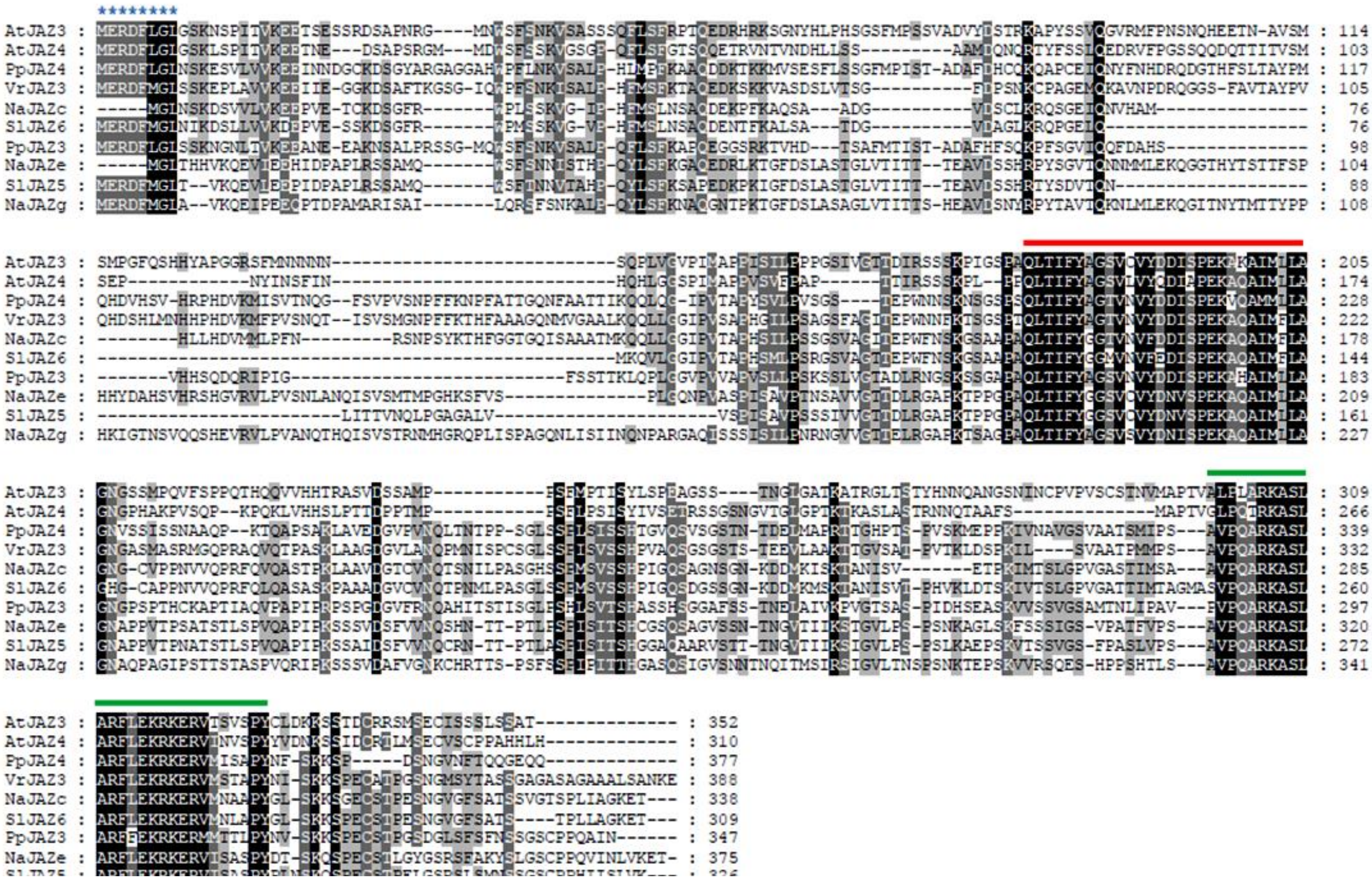

"Class III"

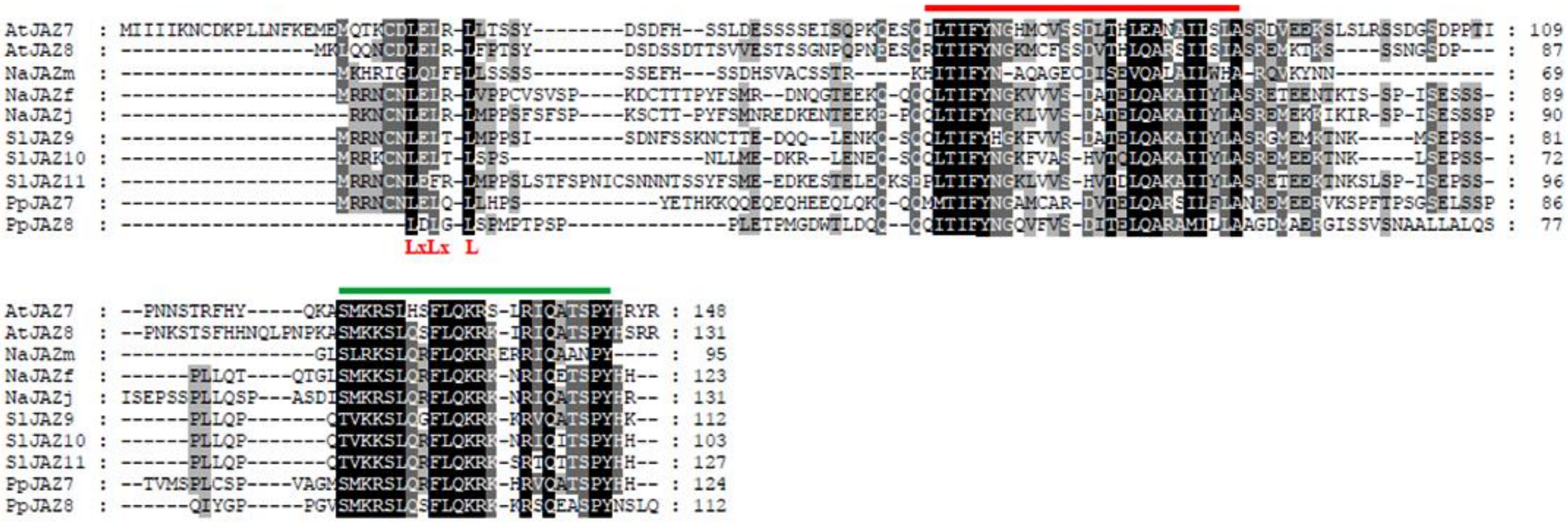

"Class IV"

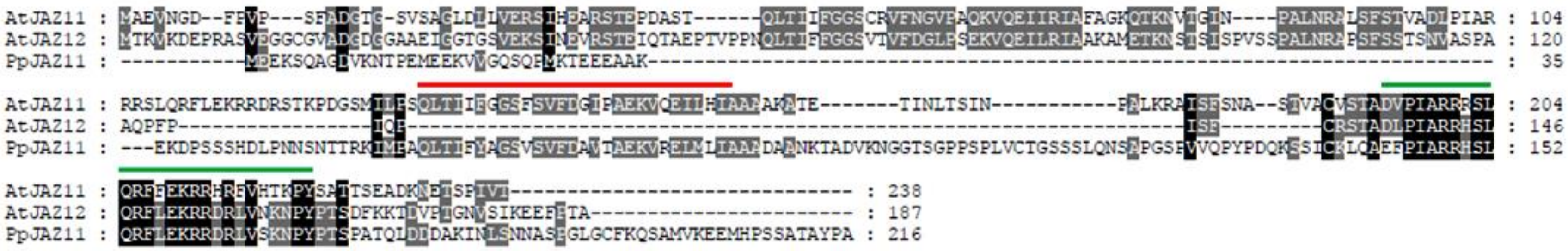

“Class V”

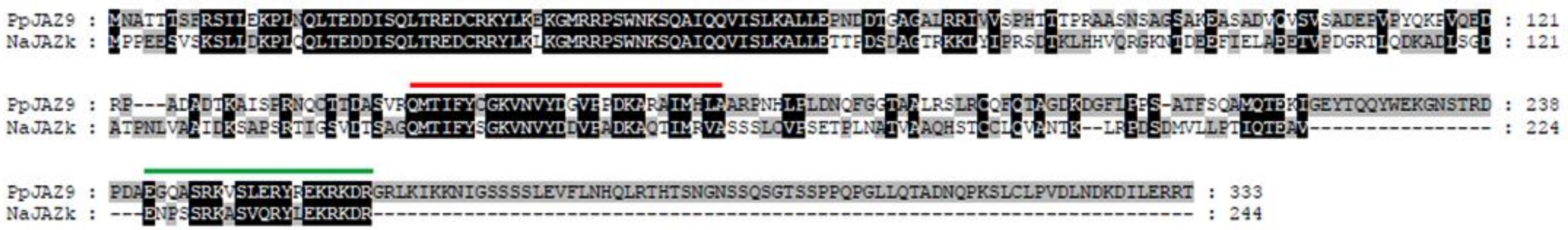

“Class VI”

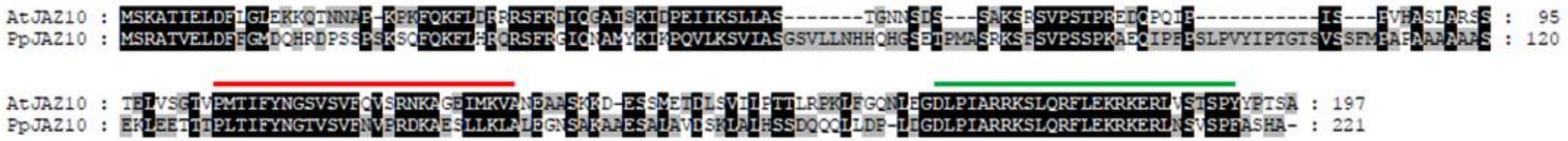

“Class VII”

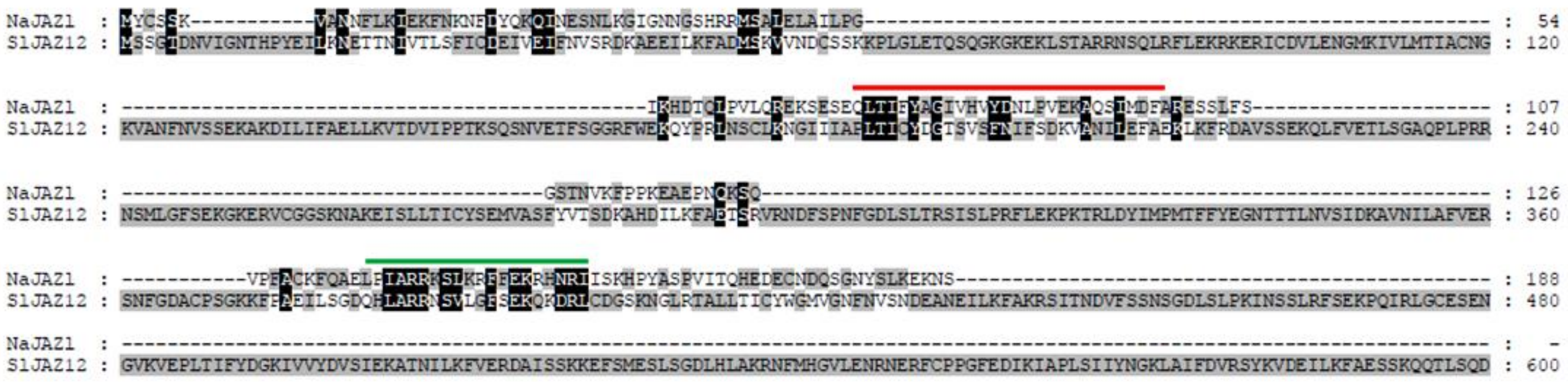

Text S1: **Multiple sequence alignments identified seven distinct classes of peach JAZ proteins.** Deduced amino acid sequences of JAZ proteins from peach and other plant species were aligned using Clustal X and edited using GeneDoc. The two characteristic motifs, ZIM and Jas, were identified for all JAZ proteins and pointed by red and green solid lines, respectively. A weakly conserved N-terminal (NT) motif was identified in Class I -JAZs and indicated by a blue solid line. A highly conserved motif was identified in Class II -JAZs and indicated by stars. An EAR-like element was identified at the N-terminus of Class III -JAZs and was indicated by red letters.
