# Supplementary material for: A stable JAZ protein from peach mediates the transition from outcrossing to self-pollination
Source: BMC Biol. 2015 Feb 13;13:11. doi: 10.1186/s12915-015-0124-6 (PMC4364584; doi:10.1186/s12915-015-0124-6)
Supplement: Additional file 4: Figure S3. — Phylogenetic analysis of peach JAZ proteins. Phylogenetic tree was constructed based on the available sequences for Arabidopsis thaliana (At), Nicotiana attenuata (Na), Solanum lycopersicum (Sl), Vitis rupestris (Vr), Nicotine tabacum (Nt) along with Prunus persica (Pp) JAZ proteins. PpJAZ proteins are indicated by black circles in the phylogram. [file 12915_2015_124_MOESM4_ESM.pdf]

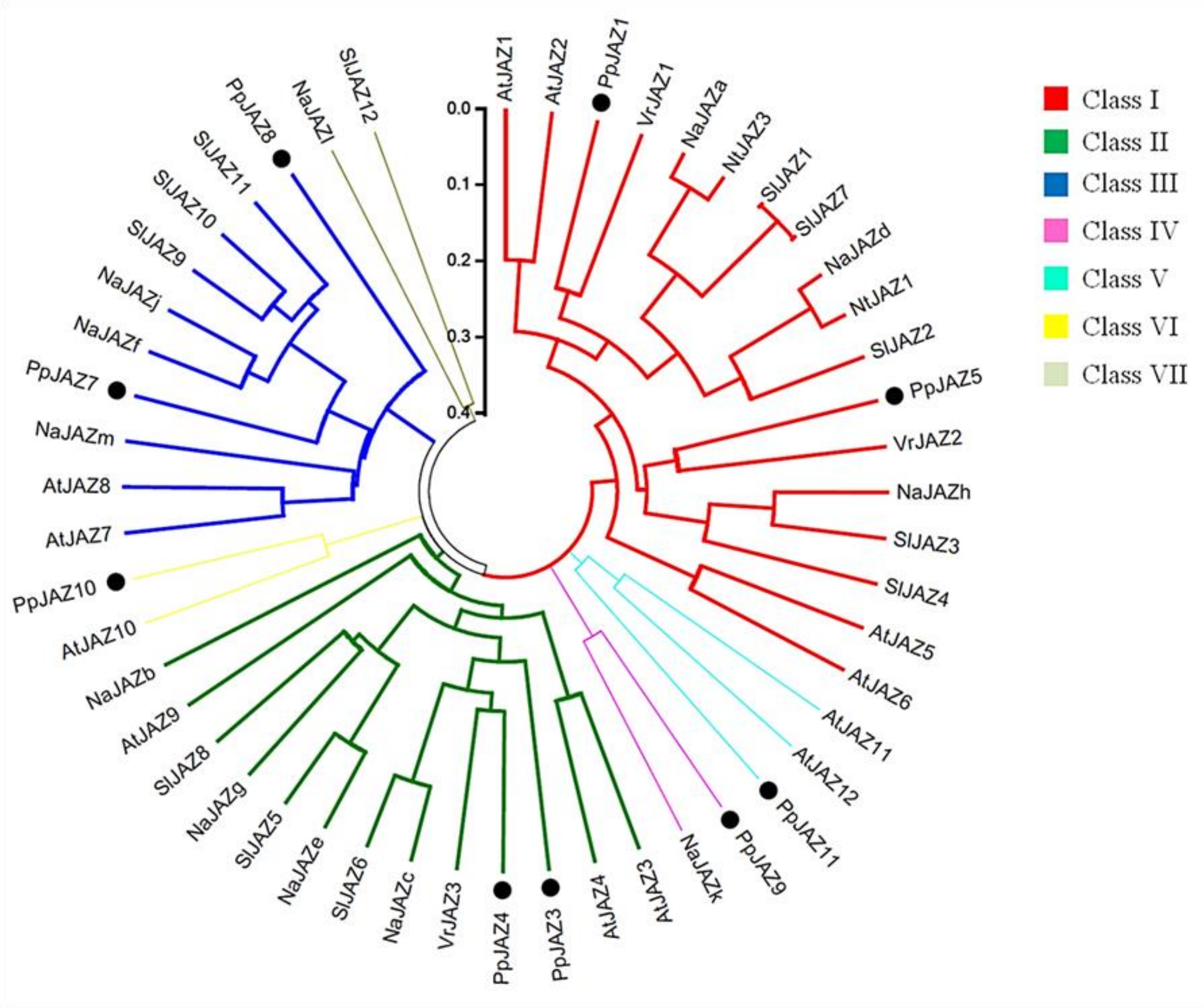

Figure S3: Phylogenetic analysis of peach JAZ proteins. Phylogenetic tree was constructed based on the available sequences for *Arabidopsis thaliana* (*At*), *Nicotiana attenuate* (*Na*), *Solanum lycopersicum* (*Sl*), *Vitis rupestris* (*Vr*), *Nicotine tabacum* (*Nt*) along with *Prunus persica* (*Pp*) JAZ proteins. PpJAZ proteins was indicates by black circles in the phylogram.
