# Supplementary material for: A stable JAZ protein from peach mediates the transition from outcrossing to self-pollination
Source: BMC Biol. 2015 Feb 13;13:11. doi: 10.1186/s12915-015-0124-6 (PMC4364584; doi:10.1186/s12915-015-0124-6)
Supplement: Additional file 5: — Text S2. PpJAZ1 promoter sequences isolated from different peach varieties. The 1,226 bp sequence upstream the translational start site of PpJAZ1 was isolated from the genomic library of ‘VABM29’, ‘Bounty’, ‘Glowing Star’ and ‘V85331’ peach varieties. Alignment of promoter sequences using Clustal X identified six SNPs among peach varieties. Further analysis of promoter sequences using PlantPAN web tool showed that one of these SNPs is located within the binding site of AINTEGUMENTA transcription factor as indicated by the red stars. [file 12915_2015_124_MOESM5_ESM.pdf]

```
Bounty       : ATTTTCTTTACTATTTTTTGAATATAACCTTTTAAACACTCACTGCAACACTCATTGCAAACCATGAGACTCATATGCCAATTAAAAATAAAAAAAAATATGGCAATTAATGCATTA
V85331       : ATTTTCTTTACTATTTTTTGAATATAACCTTTTAAACACTCACTGCAACACTCATTGCAAACCATGAGACTCATATGCCAATTAAAAATAAAAAAAAATATGGCAATTAATGCATTA
Glowing Star : ATTTTCTTTACTATTTTTTGAATATAACCTTTTAAACACTCACTGCAACACTCATTGCAAACCATGAGACTCATATGCCAATTAAAAATAAAAAAAAATATGGCAATTAATGCATTA
VABM29       : ATTTTCTTTACTATTTTTTGAATATAACCTTTTAAACACTCACTGCAACACTCATTGCAAACCATGAGACTCATATGCCAATTAAAAATAAAAAAAAATATGGCAATTAATGCATTA

Bounty       : TGCATGCATTGCAATAAGTATGGGACCTACAACTCTATTGAATCGCATTATACCTTTCTTTGCCTATATTTTTGAAAAAAAAAAAATTCAAATATATACATTTCCATATAATATTAAA
V85331       : TGCATGCATTGCAATAAGTATGGGACCTACAACTCTATTGAATCGCATTATACCTTTCTTTGCCTATATTTTTGAAAAAAAAAAAATTCAAATATATACATTTCCATATAATATTAAA
Glowing Star : TGCATGCATTGCAATAAGTATGGGACCTACAACTCTATTGAATCGCATTATACCTTTCTTTGCCTATATTTTTGAAAAAAAAAAAATTCAAATATATACATTTCCATATAATATTAAA
VABM29       : TGCATGCATTGCAATAAGTATGGGACCTACAACTCTATTGAATCGCATTATACCTTTCTTTGCCTATATTTTTGAAAAAAAAAAAATTCAAATATATACATTTCCATATAATATTAAA

Bounty       : AATTGATTGTTTTTAAAGAATGGTTTTTGAGAATATTTCAATGATACAATTTTAAGAAGATAACTACTAATTTGAAGCTATTAAATTGTACCATTGCAAATACAAATACAATTAATT
V85331       : AATTGATTGTTTTTAAAGAATGGTTTTTGAGAATATTTCAATGATACAATTTTAAGAAGATAACTACTAATTTGAAGCTATTAAATTGTACCATTGCAAATACAAATACAATTAATT
Glowing Star : AATTGATTGTTTTTAAAGAATGGTTTTTGAGAATATTTCAATGATACAATTTTAAGAAGATAACTACTAATTTGAAGCTATTAAATTGTACCATTGCAAATACAAATACAATTAATT
VABM29       : AATTGATTGTTTTTAAAGAATGGTTTTTGAGAATATTTCAATGATACAATTTTAAGAAGATAACTACTAATTTGAAGCTATTAAATTGTACCATTGCAAATACAAATACAATTAATT

Bounty       : TCAATATATGTCAAAATAATTCAATCAATTCAACTATTGAACAACCATGATAGATGCTCTTAAAACGATAATTTAGGTCTGATTTTATCAATTTGAAACACATCTTAATTTGTTTTC
V85331       : TCAATATATGTCAAAATAATTCAATCAATTCAACTATTGAACAACCATGATAGATGCTCTTAAAACGATAATTTAGGTCTGATTTTATCAATTTGAAACACATCTTAATTTGTTTTC
Glowing Star : TCAATATATGTCAAAATAATTCAATCAATTCAACTATTGAACAACCATGATAGATGCTCTTAAAACGATAATTTAGGTCTGATTTTATCAATTTGAAACACATCTTAATTTGTTTTC
VABM29       : TCAATATATGTCAAAATAATTCAATCAATTCAACTATTGAACAACCATGATAGATGCTCTTAAAACGATAATTTAGGTCTGATTTTATCAATTTGAAACACATCTTAATTTGTTTTC

Bounty       : CAGTCTCTTCTATTGGTATCAACCTTATAAGTTACTAAAAAAACAAATAAGAAAAAGAAAAATAGGCAGATATTATATATTATTATTATAAATAAATAAAAAGAACAGAATGACGTG
V85331       : CAGTCTCTTCTATTGGTATCAACCTTATAAGTTACTAAAAAAACAAATAAGAAAAAGAAAAATAGGCAGATATTATATATTATTATTATAAATAAATAAAAAGAACAGAATGACGTG
Glowing Star : CAGTCTCTTCTATTGGTATCAACCTTATAAGTTACTAAAAAAACAAATAAGAAAAAGAAAAATAGGCAGATATTATATATTATTATTATAAATAAATAAAAAGAACAGAATGACGTG
VABM29       : CAGTCTCTTCTATTGGTATCAACCTTATAAGTTACTAAAAAAACAAATAAGAAAAAGAAAAATAGGCAGATATTATATATTATTATTATAAATAAATAAAAAGAACAGAATGACGTG

Bounty       : CACGCGAAACAACATGGGGCTAAGAAAAATAAACATGAACATTCTGGTGGGGAAGAAAATAATATAATAAAATAGGCGGAGGTGATACTGTAAATAAGTTGAAAATGCAGGGGTAA
V85331       : CACGCGAAACAACATGGGGCTAAGAAAAATAAACATGAACATTCTGGTGGGGAAGAAAATAATATAATAAAATAGGCGGAGGTGATACTGTAAATAAGTTGAAAATGCAGGGGTAA
Glowing Star : CACGCGAAACAACATGGGGCTAAGAAAAATAAACATGAACATTCTGGTGGGGAAGAAAATAATATAATAAAATAGGCGGAGGTGATACTGTAAATAAGTTGAAAATGCAGGGGTAA
VABM29       : CACGCGAAACAACATGGGGCTAAGAAAAATAAACATGAACATTCTGGTGGGGAAGAAAATAATATAATAAAATAGGCGGAGGTGATACTGTAAATAAGTTGAAAATGCAGGGGTAA
                                                                                   ****************
Bounty       : CGTTGAAGTTGGCAGATGGGAACGCAAACTTGGACGTTACCAAGCGATGCAAAGAACACGTGTTTGAAACCTGAAACCGACGGGTGGTAAGGCCCACCGGGACAAATTTTTTTAAAA
V85331       : CGTTGAAGTTGGCAGATGGGAACGCAAACTTGGACGTTACCAAGCGATGCAAAGAACACGTGTTTGAAACCTGAAACCGACGGGTGGTAAGGCCCACCGGGACAAATTTTTTTAAAA
Glowing Star : CGTTGAAGTTGGCAGATGGGAACGCAAACTTGGACGTTACCAAGCGATGCAAAGAACACGTGTTTGAAACCTGAAACCGACGGGTGGTAAGGCCCACCGGGACAAATTTTTTTAAAA
VABM29       : CGTTGAAGTTGGCAGATGGGAACGCAAACTTGGACGTTACCACNCGATGCAAAGAACACGTGTTTGAAACCCGAAACCGACGGGTGGTAAGGCCCCCCGGGACAAATTTTTTTAAAA

Bounty       : ATAAACATCACGTAGACCTCGGAA-GGTGAGAGGAACCCGCCTTTTAAAAACACCCATATATTCCTCGTGCTTTAACTCAAACCCCACTAATAATTAAAAACCACTAAATAAACACA
V85331       : ATAAACATCACGTAGACCTCGGAA-GGTGAGAGGAACCCGCCTTTTAAAA-CACCCATATATTCCTCGTGCTTTAACTCAAACCCCACTAATAATTAAAAACCACTAAATAAACACA
Glowing Star : ATAAACATCACGTAGACCTCGGAAAGGTGAGAGGAACCCGCCTTTTAAAAACACCCATATATTCCTCGTGCTTTAACTCAAACCCCACTAATAATTAAAAACCACTAAATAAACACN
VABM29       : ATAAACATCACGTAGACCTCGGAA-GGGGAGAGGAACCCGCCTTTTAAAAACACCCATATATTCCTCGTGCTTTAACTCAAACCCCACTAATAATTAAAAACCACTAAATAAACACA

Bounty       : TAATATTTTATTAAATTAATAAGCAGAGATAAGCCTCTCTCTCTCTCTCTCTCTCTCTCTCTCAGAGGAAGATGTAGATAAAGCCATAAGGGGTAGAGAGAGAGGAGAGAGGG
V85331       : TAATATTTTATTAAATTAATAAGCAGAGATAAGCCTCTCTCTCTCTCTCTCTCTCTCTCTCTCAGAGGAAGATGTAGATAAAGCCATAAGGGGTAGAGAGAGAGGAGAGAGGG
Glowing Star : TAATATTTTATTAAATTAATAAGCAGAGATAAGCCTCTCTCTCTCTCTCTCTCTCTCTCTCTCAGAGGAAGATGTAGATAAAGCCATAAGGGGTAGAGAGAGAGGAGAGAGGG
VABM29       : TAATATTTTATTAAATTAATAAGCAGAGATAAGCCTCTCTCTCTCTCTCTCTCTCTCTCTCTCAGAGGAAGATGTAGATAAAGCCATAAGGGGTAGAGAGAGAGGAGAGAGGG

Bounty       : CTATAAGTCTTCTCTCAGTACATGAAGAAAAGAGTATAAAGAGAGCTTGTTTCAGCGCCAAGACTGATACTGCCATATTTGTCTGTCTTTATTTTTATATTTTTGCGTCTAGAAAAG
V85331       : CTATAAGTCTTCTCTCAGTACATGAAGAAAAGAGTATAAAGAGAGCTTGTTTCAGCGCCAAGACTGATACTGCCATATTTGTCTGTCTTTATTTTTATATTTTTGCGTCTAGAAAAG
Glowing Star : CTATAAGTCTTCTCTCAGTACATGAAGAAAAGAGTATAAAGAGAGCTTGTTTCAGCGCCAAGACTGATACTGCCATATTTGTCTGTCTTTATTTTTATATTTTTGCGTCTAGAAAAG
VABM29       : CTATAAGTCTTCTCTCAGTACATGAAGAAAAGAGTATAAAGAGAGCTTGTTTCAGCGCCAAGACTGATACTGCCATATTTGTCTGTCTTTATTTTTATATTTTTGCGTCTAGAAAAG

Bounty       : GGTAAAAAAAATTGGATTTTGAGTTGTGGGTTGTGTGATTGTGAATTGGGTCGGCGAGAATG : 1230
V85331       : GGTAAAAAAAATTGGATTTTGAGTTGTGGGTTGTGTGATTGTGAATTGGGTCGGCGAGAATG : 1229
Glowing Star : GGTAAAAAAAATTGGATTTTGAGTTGTGGGTTGTGTGATTGTGAATTGGGTCGGCGAGAATG : 1231
VABM29       : GGTAAAAAAAATTGGATTTTGAGTTGTGGGTTGTGTGATTGTGAATTGGGTCGGCGAGAATG : 1230
```

Text S2: *PpJAZ1* promoter sequences isolated from different peach varieties. The 1226 bp sequence upstream the translational start site of *PpJAZ1* was isolated from the genomic library of ‘VABM29’, ‘Bounty’, ‘Glowing Star’ and ‘V85331’ peach varieties. Alignment of promoter sequences using Clustal X identified four SNPs and two INDELs among peach varieties. Further analysis of promoter sequences using PlantPAN web tool showed that one of these INDELs is located within the binding site of AINTEGUMENTA transcription factor as pointed by the red stars.
