# Supplementary material for: A stable JAZ protein from peach mediates the transition from outcrossing to self-pollination
Source: BMC Biol. 2015 Feb 13;13:11. doi: 10.1186/s12915-015-0124-6 (PMC4364584; doi:10.1186/s12915-015-0124-6)
Supplement: Additional file 6: Table S1. — cis-regulatory elements identified in PpJAZ1 promoter sequence. Table S2. Phenotypic characterization of PpJAZ1 tobacco lines. Table S3. Differential gene expression in leaves of transgenic tobacco plants. Table S4. Differential gene expression in petals of transgenic tobacco plants. Table S5. Sequences of the oligonucleotides described in Methods. Table S6. Accession numbers for genes used in this study. [file 12915_2015_124_MOESM6_ESM.pdf]

Table S1: *cis*-regulatory elements identified in *PpJAZ1* promoter sequence.

Putative *cis*-element / consensus	Putative function/response	Strand	Location	Reference
**B-Myb / ACCWACCNN**	MYB-homologous P gene controls phlobaphene pigmentation in maize floral organs.	-	802	Grotewold et al., 1994
**MYB.Ph3 / AWAAAACSGTTAY & NRRAGTTAGTTAS**	A petunia petal epidermis-specific MYB transcription factor (MYB.Ph3)	-	548, 67, 154, 704	Solano et al., 1995
**AGL3 / NNTNCCAWWWATAGNWNN**	Arabidopsis MADS-box gene similar to floral homeotic gene AGAMOUS	+	642, 792, 894	Hang et al., 1995
**ZF-HD / ATTA**	Arabidopsis zinc finger-homeodomain genes encode proteins coordinately expressed during floral development.	-	75, 121, 142, 195, 232, 422, 427, 690, 716, 732	Tan and Irish, 2006
		+	2,88, 119, 316, 323, 326, 329, 692, 725, 730	
**POLLEN1LELAT52 / AGAAA**	One of two co-dependent regulatory elements responsible for pollen specific activation of tomato lat52 gene.	+	294, 300, 385, 416, 859, 945	Bate and Twell (1998)

Table S2: Phenotypic characterization of *PpJAZ1* tobacco lines.

Plant ID	Plant height (cm)	largest leaf width (cm)	Petal length (mm)	Pistil length (mm)	Stamen length (mm)	Weight of five fruits (gm)
**Cl1 D**	100	23	50	44	41	1.32
**Cl1 E**	86	19	58	47	48	1.45
**Cl1 F**	117	22	51	42	40	1.5
**Cl1 H**	107	21	56	45	42	1.61
**Cl1 J**	96	20	52	43	44	1.35
**Cl1 K**	86	21	54	44	46	1.1
**Cl2 E**	111	33	49	43	42	0.88
**Cl2 F**	116	24	48	40	41	0.89
**Cl2 G**	120	24	49	44	39	1.02
**Cl2 H**	102	21	49	43	43	0.92
**Cl2 I**	96	22	46	40	37	0.7
**Cl2 J**	92	22	25	19	12	NA
**Cl2 K**	110	19	52	43	43	0.98
**Cl3 A**	95	22	46	40	37	0.67
**Cl3 B**	92	23	48	40	41	0.88
**Cl3 E**	76	21	49	41	40	0.88
**Cl3 F**	77	21	51	43	40	1.1
**Cl3 G**	85	18	52	43	40	1.4
**Cl3 K**	97	21	49	44	40	0.76
**Cl3 L**	93	22	29	23	17	NA
**Cl3 M**	91	20	48	40	41	0.98
**Cl3 N**	94	22	25	20	13	NA
**Cl3 O**	90	23	30	32	13	NA
**Cl3 P**	87	23	53	41	43	1.1
**Cl3 Q**	102	20	32	22	13	NA
**WT A**	84	21	55	47	47	1.5
**WT D**	106	22	54	44	47	1.4
**WT E**	85	18	55	45	46	1.45

ND: fruit set was Not Detected in these lines.

Table S3: Differential gene expression in leaves of transgenic tobacco plants.

Plant ID	*PpJAZ1*[a]	*NtAOC*[b]	*NtOPR3*[b]	*NtLOX3*[b]	*NtARF8*[b]	*NtMYC2a*[b]	*NtMYC2b*[b]	*NtMYB21*[b]
**Cl1 D**	690.67472*	-1.21373	1.561655	-1.07412	-1.03705	-2.72436*	-3.03098	ND
**Cl1 E**	1.00000	1.464122	1.993	2.38633*	1.31600	-1.21232	-1.34624	ND
**Cl1 F**	700.74541*	-1.634223	2.869265	15.90337*	-2.75203*	4.32995*	4.16447*	ND
**Cl1 H**	1421.05085*	-5.116712*	-1.595973	1.41218	-3.46301	-1.17892	-1.07934	ND
**Cl1 J**	0.35556	-1.567531	1.843883	7.37406*	-5.50591*	2.94850*	2.97742*	ND
**Cl1 K**	1.40553	1.367676	1.297361	1.95578	-1.01465	-1.15309	1.07187	ND
**Cl2 E**	1612.52471*	-3.393874	-1.224891	-1.19282	-3.58821	-1.09711	1.03453	ND
**Cl2 F**	3312.24940	-3.68011	-1.691162	-3.96671*	-4.77883*	-2.57424*	-2.42843	ND
**Cl2 G**	2808.08552*	-2.73599	1.299783	-1.07670	-5.27574*	1.01557	-1.09244	ND
**Cl2 H**	1402.65148*	-3.823546	-1.037462	9.07497*	-1.04004	3.83652*	2.96479*	ND
**Cl2 I**	4135.69868*	-2.413135*	1.791097	2.06682*	-1.53184	1.51753	1.17222	ND
**Cl2 J**	5316.34593*	-2.826888	-1.192632	2.32422*	-2.76729	2.00377*	1.58628	ND
**Cl2 K**	1518.31832*	-2.345719	1.201961	2.45234	-4.44265*	1.50066	1.74547	ND
**Cl3 A**	1829.93605*	-1.822224	-1.475208	-2.37871*	-1.05000	-2.77927*	-2.60163	ND
**Cl3 B**	1214.51701*	-2.051565	1.658674	1.73589	-6.62515*	1.15547	1.37391	ND
**Cl3 E**	1131.32709*	-2.272063	1.36794	1.04847	-10.43240	-1.02560	1.16003	ND
**Cl3 F**	1295.32274*	-2.331655*	2.032482*	1.33412	-5.33136*	1.04550	1.25619	ND
**Cl3 G**	1509.18026*	-2.071814	-1.341194	1.20589	-1.07158	-1.69369	-1.65922	ND
**Cl3 K**	2490.82736*	-2.612904	-1.261909	1.00720	-2.12064*	-1.06094	-1.20557	ND
**Cl3 L**	5231.50306*	-2.375213	2.244356*	6.36682*	-5.47342*	1.47523	1.74606	ND
**Cl3 M**	671.82082	-3.890696	1.091036	2.52202	-4.58906*	-1.03535	1.47728	ND
**Cl3 N**	5326.21415	-2.191635	1.927552	-1.13468	-2.59430	-1.13489	-1.09359	ND
**Cl3 O**	5777.04038*	-3.144197*	1.688294	1.19597	-4.52866*	1.21028	1.21495	ND
**Cl3 P**	1100.81912*	-2.963885*	1.180272	5.30189*	-4.65681*	3.01308*	2.92596*	ND
**Cl3 Q**	6148.07681*	-6.589559*	-1.131397	1.90674	-3.17087*	1.26890	1.22179	ND
**WT A**	ND	1.244577	1.042466	1.74274	1.02348	-1.31069	-1.59769	ND
**WT D**	ND	1.626388	1.777516	1.88989	1.11596	1.04742	-1.35804	ND
**WT E**	ND	1.00000	1.00000	1.00000	1.00000	1.00000	1.00000	ND

[a]Expression of the gene was calculated relative to that in the Cl1 E and normalized to the expression of *NtActin* in the same cDNA sample.

[b]Expression of the gene was calculated relative to that in wild-type E (WT E) and normalized to the expression of *NtActin* in the same cDNA sample.

[*] Expression of the gene is significantly up-regulated or down-regulated (minus values) compared to control at $P \leq 0.01$.

ND: The expression of the gene is Not Detected.

Table S4: Differential gene expression in petals of transgenic tobacco plants.

Plant ID	*PpJAZ1*[a]	*NtAOC*[b]	*NtOPR3*[b]	*NtLOX3*[b]	*NtARF8*[b]	*NtMYC2a*[b]	*NtMYC2b*[b]	*NtMYB21*[b]
**Cl1 D**	599.47983*	-5.22034	-1.04495	-1.19985	-1.95373	-1.03325	-1.57559	1.96488
**Cl1 E**	1.00000	1.03966	2.55533	-1.15119	-1.55422	1.93781	-1.85905	1.06842
**Cl1 F**	615.80330*	-2.03043	1.04409	-1.53887	-2.51126	1.04740	-2.14643*	1.55885
**Cl1 H**	1230.16859*	-6.81727	1.20778	1.39409	-1.93650	1.31495	-1.15117	-3.15013*
**Cl1 J**	0.30876	-1.26778	2.22086	-11.08766*	1.07095	-2.88124	-4.03496	1.59113
**Cl1 K**	1.21995	-1.31966	2.40599	1.13634	-1.24747	1.83660	-1.37775	1.33955
**Cl2 E**	854.91371	1.26421	1.86393	-1.20919	-1.76881	-1.10852	-1.95497	-2.74657*
**Cl2 F**	2863.46732*	2.22378	1.97825	-1.61257	-2.21535	-1.03878	-2.52522*	-3.44276*
**Cl2 G**	2442.32862*	-1.03355	1.93910	-1.47595	1.86614	-1.48686	-1.58710	-3.68545*
**Cl2 H**	1255.58613*	-4.19602	2.22473	-1.03904	-2.81720	-1.03842	-1.39469	1.62703
**Cl2 I**	3588.21551*	1.55430	2.66975	-1.15631	-1.56621	-1.09845	-1.73896	-12.03814*
**Cl2 J**	4732.61125*	2.00511	2.10318	-1.53569	-2.04577	-1.06733	-2.41828*	-15.81608*
**Cl2 K**	808.08845	-2.82351	1.60236	1.26938	-2.83710	1.69944	1.02800	1.27402
**Cl3 A**	1588.16966*	2.37078	2.33308	-5.37657*	1.90543	-2.63565	-4.37952*	-13.54573*
**Cl3 B**	1066.39716*	2.07681	2.46043	-5.17523*	2.27172	-2.61036	-3.95036*	-12.57171*
**Cl3 E**	979.02175*	-3.90144	1.49131	1.64444	-2.23993	1.55724	1.33677	1.60722
**Cl3 F**	1119.49588*	-1.93526	2.39136	-1.32984	-3.54435	1.04341	-1.77648	1.34819
**Cl3 G**	1348.39229*	-5.38432	1.28014	1.03305	-2.46277	1.31106	-1.54008	-4.04402*
**Cl3 K**	1758.17310*	-1.23152	1.88463	-1.56888	1.63106	-1.50880	-1.66759	-4.17453*
**Cl3 L**	3636.42800*	8.41848	2.77750	-22.04569*	24.70229*	-10.86203	-11.99998*	ND
**Cl3 M**	590.78114*	-1.93488	2.91037	-1.01084	-1.10503	1.00144	-1.01627	1.75106
**Cl3 N**	3111.88700	5.73896*	2.21125	-30.54486*	18.20450*	-17.31649	-14.54614*	-802.41034*
**Cl3 O**	5013.29303*	7.06130	2.66291	-23.53369*	20.76324*	-10.93409	-12.91132*	ND
**Cl3 P**	705.21586*	-2.17828	2.88500	-1.05159	-1.25967	1.01229	-1.08128	1.45539
**Cl3 Q**	3683.03502*	4.57435*	2.06570	-35.04265*	15.09266*	-18.07475	-15.79126*	-865.89041*
**WT A**	ND	-2.65137	1.38170	-24.10477*	-1.00448	-6.82702*	-8.19680*	1.45040
**WT D**	ND	-5.62415	2.14366	-12.14198*	-1.13360	-2.83655	-4.23817*	1.31867
**WT E**	ND	1.00000	1.00000	1.00000	1.00000	1.00000	1.00000	1.00000

[a]Expression of the gene was calculated relative to that in the Cl1 E and normalized to the expression of *NtActin* in the same cDNA sample.

[b]Expression of the gene was calculated relative to that in wild-type E (WT E) and normalized to the expression of *NtActin* in the same cDNA sample.

[*] Expression of the gene is significantly up-regulated or down-regulated (minus values) compared to control at $P \leq 0.01$.

ND: The expression of the gene is Not Detected.

Table S5: Sequences of the oligonucleotides described in Methods

Primers for qRT-PCR	
PpLOX3-RT-F	AGCCAGGGTGTCGAGAGTT
PpLOX3-RT-R	ACCAGTGGCTGATGAGCTG
PpOPR3-RT-F	GATGCGTCTCATGCCACTT
PpOPR3-RT-R	CAAGGCAGCCTGTCGATAA
PpActin-RT-F	CATGCCATTCTTCGTCTGG
PpActin-RT-R	TCAGCAGTGGTGGTGAACA
PpJAZ1-RT-F	CTCAACCCATTGTTTGTGAT
PpJAZ1-RT-R	GGTATGGTGCTTTGTTGTT
PpJAZ10-RT-F	TCCTTCTCAACCACCACCA
PpJAZ10-RT-R	TGGGATTTGCTCTGCTTTG
PpJAZ3-RT-F	TCAGGGATGCAGTGGTCAT
PpJAZ3-RT-R	TTCTTGACCCACCTTCTTGG
PpJAZ4-RT-F	CGATAGGCAAGATGGCACT
PpJAZ4-RT-R	TTCACATCATGGGGACGAT
PpJAZ5-RT-F	AACATGCAACCTCCTGAGC
PpJAZ5-RT-R	GGCCGGTCTCCATGTTAGT
PpJAZ11-RT-F	TTCGCTGCAAAACTCTGCT
PpJAZ11-RT-R	TGGGAAATTCAGCTTGCAG
PpJAZ7-RT-F	GCGCTCGTGATGTTACAGA
PpJAZ7-RT-R	TCCACTTGGAGTGAATGGAC
PpJAZ8-RT-F	TATGGCAGAAAGGGGGATA
PpJAZ8-RT-R	GACCTCTTCATGGACACACC
PpJAZ9-RT-F	CTGCCGCAAATACCTCAAA
PpJAZ9-RT-R	AGCAGAGCCTTGAGGGAAA
NtActin-F	GTTGCTATTCAGGCTGTCCTTT
NtActin-R	CATCACCAGAATCCAACACAA
NtAOC-RT-F	TGATCAAACACGAAGAGGAAA
NtAOC-RT-R	GAACGGCGATGTGACCATAA
NtLOX3-RT-F	GGTTTTCCCACCAGTAAGCAA
NtLOX3-RT-R	GCCATTGAGATGACCAAGAA
NtOPR3-RT-F	GCCAGATGGAACTCATGCTAT
NtOPR3-RT-R	GCGATAATCTTCAACCACTTGT
NtARF8-RT-F	TCAGCTCCTTCTGGGGATT
NtARF8-RT-R	GCCCAATGTGCATGCTATC
NtMYC2a-RT-F	CAGCTCAAGCCCGATTTGG
NtMYC2a-RT-R	CTGAAGCCCGAAACCTTGG
NtMYC2b-RT-F	GCCTGAGTCTGGCGAAATC
NtMYC2b-RT-R	TTGGCCCGAAAACAAGCTC
NtMYB21-RT-F	TATCTCCGGCCTGATGTCC
NtMYB21-RT-R	GACCACCTGTTTCCCCACT
**Primers to generate PpJAZ1-GFP construct**	

PpJAZ1.F	GCGGATCCATGTCGAGTTCATCGGAGACT
PpJAZ1.R	ATGGATCCCTTGGGTTGGTTGAGCAGCCAA
**Primers for Y2H assays**	
PpCOI1-BD-F	ATCCATGGAGATGGAGGATCGAAACGTGCGAA
PpCOI1-BD-R	ATGTCGACGGAGGTAATAAAAGATGCTGGAT
AtCOI1-BD-F	ATCCATGGAGATGGAGGATCCTGATATCAAGA
AtCOI1-BD-R	ATGTCGACTATTGGCTCCTTCAGGACTCTAAC
PpJAZ1-AD-F	ATCATATGATGTCGAGTTCATCGGAGACTCTG
PpJAZ1-AD-R	CCATCGATTTGGGTTGGTTGAGCAGCCAAG
AtJAZ1-AC-F	ATCATATGATGTCGAGTTCTATGGAATGTTCT
AtJAZ1-AC-R	CCATCGATTATTTCAGCTGCTAAACCGAGC

Table S6: Accession numbers for genes used in this study.

Gene ID	Accession #
PpActin	BU046508
PpJAZ1	EMJ03624
PpJAZ3	EMJ10481
PpJAZ4	EMJ24447
PpJAZ5	EMJ17157
PpJAZ7	EMJ13436
PpJAZ8	EMJ18496
PpJAZ9	EMJ23765
PpJAZ10	EMJ25153
PpJAZ11	EMJ03625
PpCOI1	EMJ21634
PpLOX3	EMJ18259
PpOPR3	EMJ05582
NtActin	BAD27408
NtAOC	CAC83765
NtLOX3	AY775034
NtOPR3	HQ891853
NtMYC2a	ADH04269
NtMYC2b	ADH04270
