# Supplementary material for: A stable JAZ protein from peach mediates the transition from outcrossing to self-pollination
Source: BMC Biol. 2015 Feb 13;13:11. doi: 10.1186/s12915-015-0124-6 (PMC4364584; doi:10.1186/s12915-015-0124-6)
Supplement: Additional file 8: Figure S4. — The uniformity of β-Actin expression in different samples. The qRT-PCR amplification plot for PpActin, PpJAZ5 (a), NtActin and NtMYB21 (b) in cDNAs generated from peach and tobacco flowers, respectively. The X-axis shows the number of PCR cycles and the Y-axis shows the relative fluorescent units (RFU). The amplification plot was generated for six cDNA samples and three technical replicates for each sample. [file 12915_2015_124_MOESM8_ESM.pdf]

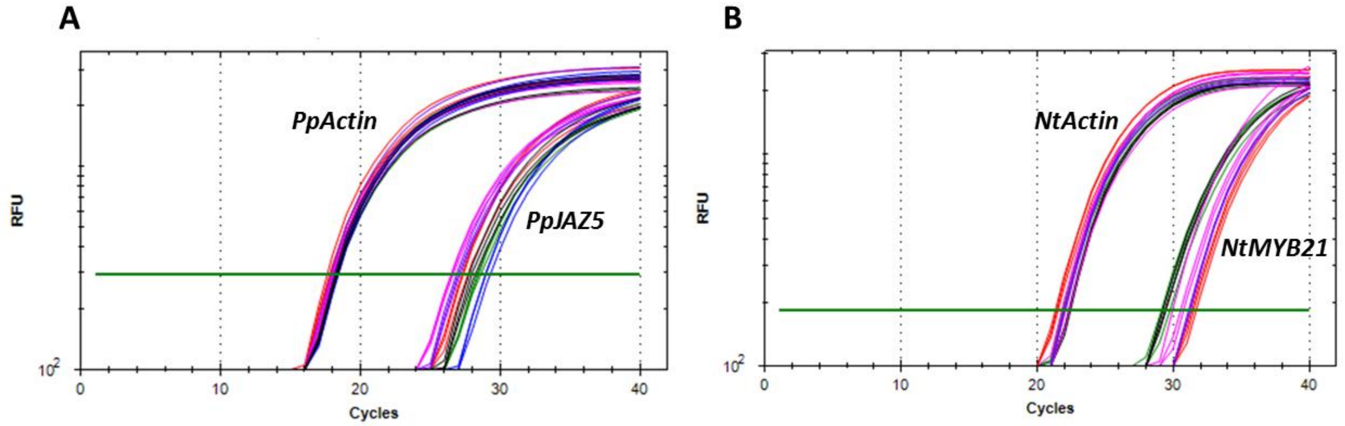

Figure S4: The uniformity of *β-Actin* expression in different samples. The qRT-PCR amplification plot for *PpActin*, *PpJAZ5* (a), *NtActin* and *NtMYB21* (b) in cDNAs generated from peach and tobacco flowers, respectively. X-axis shows the number of PCR cycles and the Y-axis shows the relative fluorescent units (RFU). The amplification plot was generated for six cDNA samples and three technical replicates for each sample.
